# The Methodological Trends of Traditional Herbal Medicine Employing Network Pharmacology

**DOI:** 10.3390/biom9080362

**Published:** 2019-08-13

**Authors:** Won-Yung Lee, Choong-Yeol Lee, Youn-Sub Kim, Chang-Eop Kim

**Affiliations:** 1 Department of Physiology, College of Korean Medicine, Gachon University, Seongnam 13120, Korea; 2 Department of Anatomy-Pointology, College of Korean Medicine, Gachon University, Seongnam 13120, Korea

**Keywords:** network pharmacology, traditional herbal medicine, methodological trend

## Abstract

Natural products, including traditional herbal medicine (THM), are known to exert their therapeutic effects by acting on multiple targets, so researchers have employed network pharmacology methods to decipher the potential mechanisms of THM. To conduct THM-network pharmacology (THM-NP) studies, researchers have employed different tools and databases for constructing and analyzing herb–compound–target networks. In this study, we attempted to capture the methodological trends in THM-NP research. We identified the tools and databases employed to conduct THM-NP studies and visualized their combinatorial patterns. We also constructed co-author and affiliation networks to further understand how the methodologies are employed among researchers. The results showed that the number of THM-NP studies and employed databases/tools have been dramatically increased in the last decade, and there are characteristic patterns in combining methods of each analysis step in THM-NP studies. Overall, the Traditional Chinese Medicine Systems Pharmacology Database and Analysis Platform (TCMSP) was the most frequently employed network pharmacology database in THM-NP studies. Among the processes involved in THM-NP research, the methodology for constructing a compound–target network has shown the greatest change over time. In summary, our analysis describes comprehensive methodological trends and current ideas in research design for network pharmacology researchers.

## 1. Introduction

Traditional herbal medicine (THM) has maintained the health of Asian people for thousands of years and built a unique medical system based on empirically accumulated knowledge. Billions of people around the world are taking THM daily, and the drug development field considers THM to be a source of inspiration [1,2]. The research indicates that THM’s therapeutic effects may involve various biomolecules [3]. However, due to the complexity of THM and the limitations of experimental applications, specific mechanisms of action have been fully elucidated for only a few THMs [4]. This is a major obstacle to THM’s modernization and wider application to modern healthcare.

Network pharmacology has emerged as a promising approach to accelerate drug development and elucidate the mechanisms of action of multiple target components [5]. It understands disease as a perturbation of interconnected complex biological networks and identifies the mechanisms of drug action by network topology [6,7]. The conceptual elements of network pharmacology were derived from systems biology, which can address both the connectivity and the interdependence of individual components [8]. The core idea of network pharmacology is well suited for analyzing the multi-targeted agents, so network pharmacology methods may be appropriate for identifying the complex mechanisms of THM.

**Figure 1:**
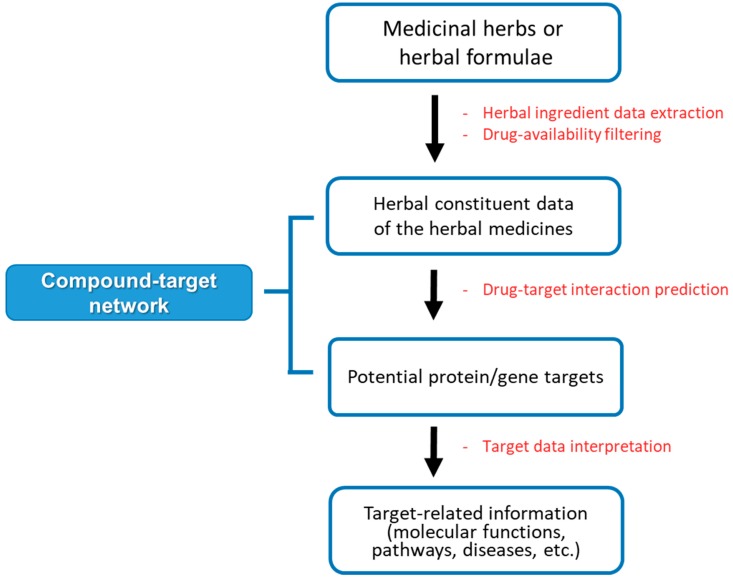
The general framework of network pharmacology analysis of herbal medicine.

In the last decade, researchers have employed network pharmacology methods to elucidate the potential targets and toxicity of THMs [9]. THM-network pharmacology (THM-NP) studies are conducted by constructing an herb–compound–target (H-C-T) network by integrating the herbal constituent data and drug-target interactions (DTIs) information. Then, the target network is analyzed to interpret related biological functions, pathways, and diseases (Figure 1). Since there are no gold standard methods for THM-NP studies yet, researchers have developed and applied various tools and databases for each step.

Several studies have described THM-NP researches by summarizing network pharmacology databases for THM and illustrating several representative applications [10,11,12,13,14,15]. Although these studies contributed to a better understanding of THM-NP studies, they were limited in providing quantitative information on the frequencies, variations, and combinatorial patterns of the employed methods in THM-NP studies. In this study, we systematically attempted to capture the methodological trends of the THM-NP research field. We identified the tools and databases employed to conduct THM-NP studies and visualized their frequency, variation, and combinatorial pattern in a step-by-step manner. The THM-NP studies were identified by searching PubMed and then preprocessed. We also constructed and analyzed the co-author and affiliation networks to identify how the diverse methods for THM-NP studies are employed and shared among researchers in the field. We believe that analyzing the methodological trends will provide a comprehensive understanding and valuable insights into the THM-NP research field.

## 2. Materials and Methods

### 2.1. Search Strategy

The literature search was performed in PubMed (https://www.ncbi.nlm.nih.gov/pubmed/) from January 2000 to December 2018. The search language was restricted to English. The search terms used were NP-related terms (“network pharmacology” OR “network analysis” OR “system-level” OR “systems-level” OR “systems pharmacology” OR “systems biology” OR “bioinformatics”) in [title] AND THM-related terms (“oriental medicine” OR “traditional medicine” OR “traditional Asian medicine” OR “Chinese medicine” OR “Kampo medicine” OR “Korean medicine”) in [title/abstract]. The search range of THM-related terms was extended to [title/abstract] since the titles of THM studies generally contain only the name of herbs or herbal formulae that are difficult to search.

### 2.2. Inclusion Criteria 

We considered a THM-NP study as the original article that analyzed a THM’s mode of action through the construction of a compound-target network. Full-text articles from the literature search were checked to determine their eligibility. There was no restriction regarding in vivo, in vitro, and in silico studies. THM was considered as (1) extract(s) from a single herb; (2) preparation(s) containing multiple herbs; (3) proprietary herbal product(s); and (4) molecule(s) derived from a single herb.

### 2.3. Study Selection and Data Extraction 

Two authors (W.Y. Lee and C.E. Kim) independently examined titles, abstracts, and journals to select eligible THM-NP studies. When articles were duplicated, only the most recent information was included. Then the full text of potentially relevant studies was retrieved. Two authors (W.Y. Lee and C.E. Kim) independently examined the full-text records to determine which studies met the inclusion criteria. Disagreements about the study selection were resolved by rechecking whether the studies met our criteria for inclusion.

Authors extracted the following data from the included THM-NP studies: authors, affiliations, publication years, tools, and databases. Synonyms for tools and databases were merged and counted under a single keyword. DTpre and SysDT [16] were considered to be the same method as Traditional Chinese Medicine Systems Pharmacology Database and Analysis Platform (TCMSP, http://lsp.nwu.edu.cn/tcmsp.php) [17] since these methods were originally developed and implemented in TCMSP. 

### 2.4. Categorizing Drug-Target Interaction Methods

To capture the trends in the methods for constructing compound-target networks, we categorized DTI methods into four groups by their hypothesis and which information was used: the chemogenomic approach, docking simulation approach, ligand-based approach, and others [18,19,20]. The chemogenomic approach predicts potential compound–target pairs similar to validated compound-target pairs. This method is based on the assumption that a compound–target pair is likely to interact with high similarity to a validated compound-target interaction in terms of chemo–physical properties [21]. The docking simulation approach predicts the binding conformation of small-molecule ligands to the appropriate binding site of the target using 3D structural information on the compounds and protein targets [22]. The key hypothesis of this approach is that compounds with a high binding affinity at the binding site are likely to interact with the target [23]. The ligand-based approach predicts interactions by comparing a new ligand to known proteins’ ligands based on the hypothesis that similar molecules usually bind to similar proteins [24]. DTI methods that do not belong to the above categories were assigned to the category “others”, such as data mining techniques, high-throughput screening, and databases that integrate drug-target interaction information from heterogeneous sources.

### 2.5. Construction of the Co-Author Network and Affiliation Network

The author network and affiliation network were constructed to identify the methodological characteristics of corresponding authors and affiliations. The nodes in each network represent authors or affiliations, and the edges represent co-occurrences of authors or affiliations in THM-NP studies. The frequencies of employed DTI and drug availability methods were counted for each corresponding author or affiliation. These methodologies were mapped to the author network and the affiliation network. Cytoscape 3.7.1 (http://www.cytoscape.org/) was used to visualize the networks [25].

## 3. Results

### 3.1. Description of the Search

**Figure 2:**
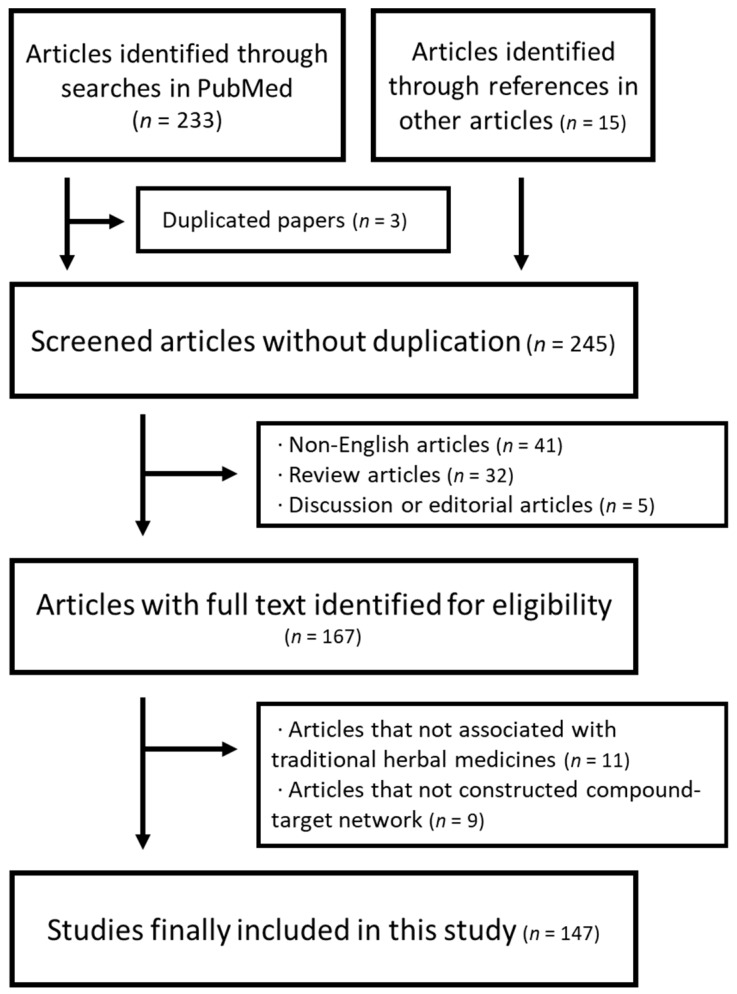
The flowchart of the study selection process.

We initially found 233 potentially relevant articles from PubMed. The search was conducted using combined keywords consisting of THM-related terms and NP-related terms. Another 15 potentially relevant articles were included by searching references in other THM-NP studies or review articles. Titles, abstracts, and journal names were screened, and 167 studies were considered potentially eligible for inclusion. Of these, 20 articles were excluded after screening the full texts. Finally, a total of 147 THM-NP studies were included in our study (Figure 2). The included THM-NP studies are listed in Supplementary Table S1.

**Figure 3:**
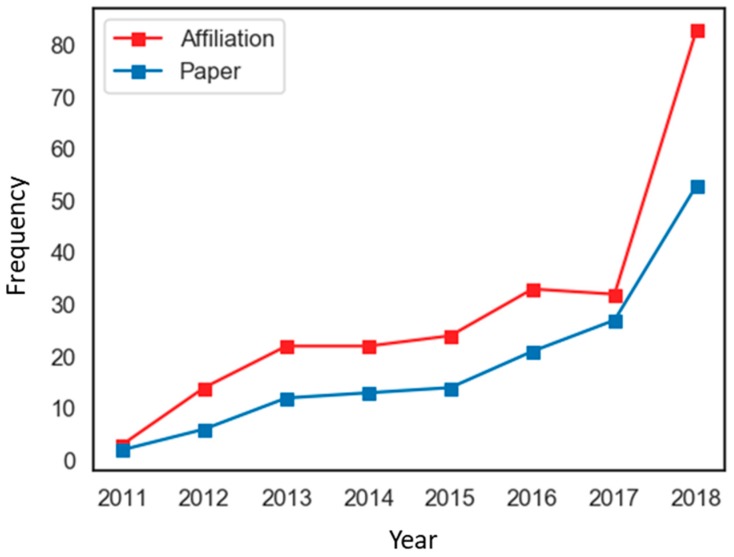
Annual publication trends of traditional herbal medicine-network pharmacology (THM-NP) studies.

We identified the number of published THM-NP studies over time, along with the affiliations involved in the studies. THM-NP studies that met our criteria for inclusion have appeared since 2011. In 2011, only three affiliations published two THM-NP studies, but in 2018, the number of affiliations and studies increased to 83 and 52, respectively (Figure 3). The increased number of papers and of affiliations involved indicates that the THM-NP research fields have continuously grown.

### 3.2. Methodological Trends in Constructing the Herb-Compound Network

**Figure 4:**
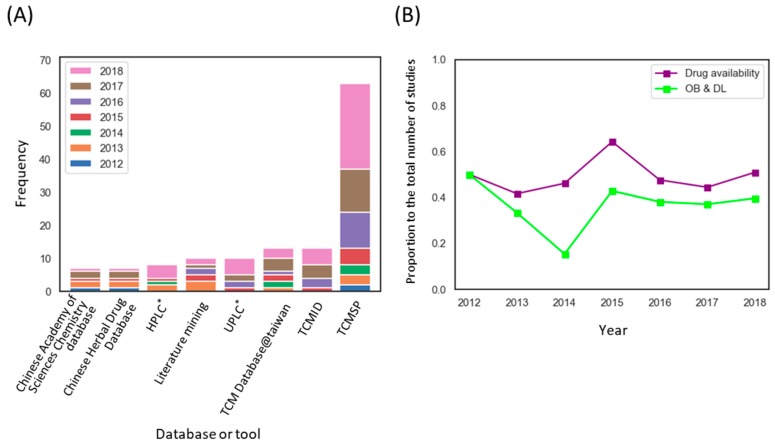
Methodological trends in the construction of the herb–compound network. (**A**) Frequency of databases and tools used to identify constituents of herbal medicine. * Analytical techniques to identify the ingredients of herbal medicines; Ultra Performance Liquid Chromatography (UPLC), High-performance liquid chromatography (HPLC) (**B**) The application rate of the drug availability method by year. Note that Obioavail (OB) and drug-likeness (DL) are among the most commonly used drug availability assessment methods in THM-NP studies. THM-NP studies in 2011 were excluded from the visualization due to the low frequency (*n* = 2). TCMSP, Traditional Chinese Medicine Systems Pharmacology Database; TCMID, Traditional Chinese Medicine Integrated Database.

We next investigated the trends in employed methods in THM-NP studies. Commonly used databases and tools are described in Table 1 (see Supplementary Table S2 for complete lists). The construction of the herb-compound network is the first step of a THM-NP study. Among the databases for herbal medicines, TCMSP was most commonly used to construct an herb-compound network. Additionally, some THM-NP researchers used their own experimental results (e.g., Ultra Performance Liquid Chromatography (UPLC) or High-performance liquid chromatography) to identify ingredients of the herbal medicines in their studies (Figure 4A).

Because information on the absorption, distribution, metabolism, and excretion (ADME) properties of herbal medicines in humans are lacking, researchers have employed evaluation methods or machine learning tools to predict those properties. We counted the number of THM-NP studies that evaluated the drug availability of herbal ingredients. We found that approximately half of (72/147, 49.0%) THM-NP studies evaluated the drug availability of herbal ingredients, and the majority of the studies (54/72, 75.0%) employed Obioavail and drug-likeness in combination (Figure 4B). Obioavail is an in silico model that predicts the fraction of an administered dose of a drug that reaches the systemic circulation unchanged [39]. Drug-likeness measures the structural similarity between herbal ingredients and the drugs in the Drugbank database (http://www.drugbank.ca/) using the Tanimoto coefficient [40]. They are applied to screen ADME-favorable compounds and pharmacologically suitable compounds in herbal medicines, respectively.

### 3.3. Methodological Trends for Constructing Compound-Target Networks

**Figure 5:**
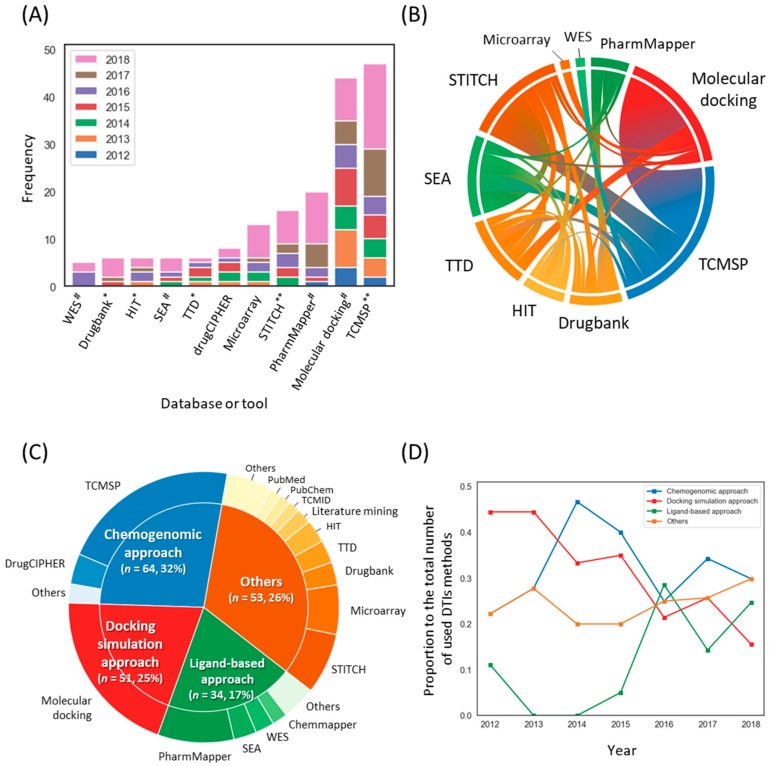
Methodological trends in the construction of the compound–target network. (**A**) The frequencies of databases and tools used to identify targets of herbal ingredients. * databases that provide validated drug-target interactions (DTIs); ** databases that provide validated databases that provide both validated and predicted DTIs; # Computational tools to predict DTIs. (**B**) A co-occurrence pattern of the DTI method. (**C**) Categories of DTI methods and their composition. The outer circle and inner circle represent the DTI methods and their categories, respectively. (**D**) The annual rate of the groups of DTI methods. Note that THM-NP studies in 2011 were excluded from the visualization due to their low frequency (*n* = 2). TCMSP, Traditional Chinese Medicine Systems Pharmacology Database; TTD, Therapeutic Target Database; SEA, Similarity Ensemble Approach; HIT, Herb Ingredients’ Targets; WES, Weighted Ensemble Similarity.

We next attempted to determine the frequency of each DTI method for constructing compound–target (C-T) networks (Note that some of the THM-NP studies combined several methods to identify DTIs. Therefore, the total frequency of the DTI method is greater than the total number of THM-NP studies). The results showed that TCMSP (47/222, 21.1%) and molecular docking (44/222, 19.8%) were the most frequently used. In addition, experimental methods, such as microarrays, were also applied (Figure 5A). It is noteworthy that DTI methods of TCMSP have existed for less than 10 years since its development but have been used most frequently in THM-NP studies [16]. More than one-third of THM-NP studies (54/147, 36.7%) combined several DTI methods for constructing C-T networks, and most of them included TCMSP (e.g., TCMSP-molecular docking and TCMSP-STITCH) (Figure 5B). 

We categorized the methods into four groups: the chemogenomic approach, docking simulation approach, ligand-based approach, and others (Figure 5C, see Materials and Methods for details). To identify trends in DTI methods, we counted the frequency of each DTI group each year (Figure 5D). In the early stage, approximately half of THM-NP studies (4/9, 44.4% and 8/18, 44.4% in 2012 and 2013, respectively) employed molecular docking simulation, but the proportion of molecular docking simulations decreased gradually and was the lowest (12/77, 15.9%) in 2018.

### 3.4. Methodological Trends for Target Interpretation

**Figure 6:**
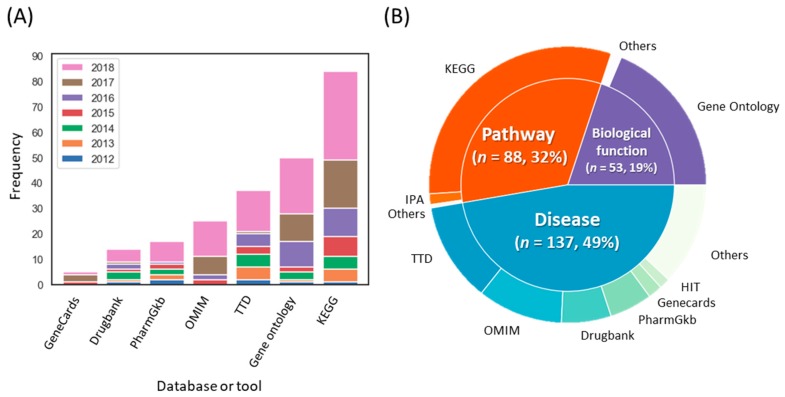
Trends in the biomedical databases used for target interpretation. (**A**) The frequencies of databases used for target interpretation. (**B**) Categories of databases and their composition. The outer circle and inner circle represent the databases used for target interpretation and their categories, respectively. Note that THM-NP studies in 2011 were excluded from the visualization due to their low frequency (*n* = 2). KEGG, Kyoto Encyclopedia of Genes and Genomes; TTD, Therapeutic Target Database; OMIM, Online Mendelian Inheritance in Man; IPA, Ingenuity Pathway Analysis.

We identified the frequency of biomedical databases employed to analyze the biological processes, pathways, and diseases from the targets of herbal medicines (Figure 6). Most THM-NP studies (94/96, 98.0%) employed single databases to analyze biological functions and pathways, such as Gene Ontology (GO, http://geneontology.org/) [35] for biological processes or the Kyoto Encyclopedia of Genes and Genomes database (KEGG, https://www.genome.jp/kegg/) [41] for pathways. On the other hand, more than half of the studies (44/76, 57.9%) integrated several databases to analyze target-related diseases, such as the Therapeutic Target Database (TTD, http://bidd.nus.edu.sg/group/cjttd/) [30], Online Mendelian Inheritance in Man (OMIM, https://www.omim.org/) [36], and Drugbank [42].

### 3.5. Combinatorial Patterns in Methodologies of THM-NP Studies

**Figure 7:**
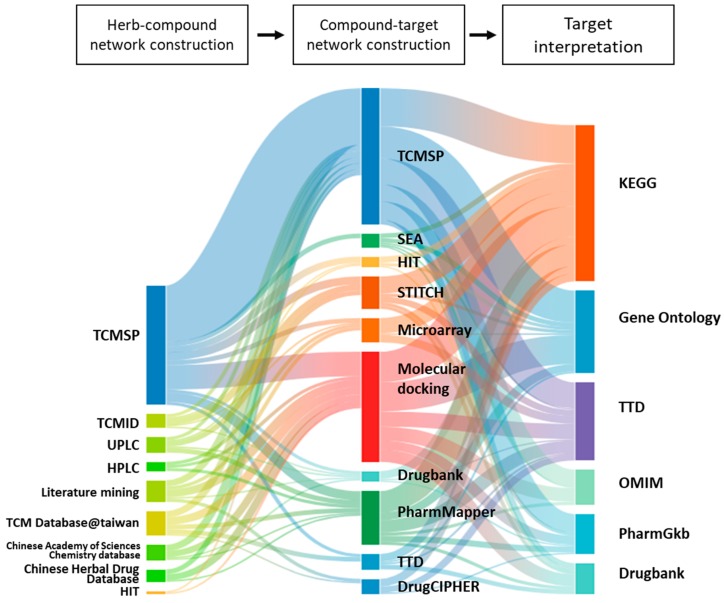
The combinatorial pattern of tools and databases employed in THM-NP studies. Each layer represents the process of network pharmacology analysis of herbal medicines, and the components of each layer represent the employed tools and databases. A connecting line between components indicates that the connected tools and databases are used together in the same THM-NP studies. The thickness of each connecting line indicates the frequency with which the two methodologies are used together in the THM-NP studies. TCMSP, Traditional Chinese Medicine Systems Pharmacology Database; TCMID, Traditional Chinese Medicine Integrated Database; HIT, Herb Ingredients’ Targets; KEGG, Kyoto Encyclopedia of Genes and Genomes; TTD, Therapeutic Target Database; OMIM, Online Mendelian Inheritance in Man.

We identified the combinatorial patterns of each step in THM-NP studies by a Sankey diagram-like representation (Figure 7). The Sankey diagram is a visualization tool used to depict quantitative information about flows from one set to another within a network [43]. The nodes in each layer (vertical lines) represent the methods of herb–compound (H-C) network construction, compound–target (C-T) network construction, and target interpretation, respectively. The edges (connected lines) between layers indicate that these methods are used together in the same THM-NP studies.

The Sankey diagram-like representation shows the diversity of databases and tools used in THM-NP studies and their combination patterns (Figure 7). We found that the nodes in the first layer (H-C network construction) tend to be connected to specific nodes in the second layer (C-T network construction), which indicates that the combinatorial pattern between the first and second layer is biased by the methods for H-C network construction. For example, TCMSP in the first layer is mainly connected to TCMSP and molecular docking in the second layer, and Traditional Chinese Medicine Integrated Database (TCMID), UPLC, and literature mining in the first layer are not linked to molecular docking in the second layer. On the other hand, the nodes in the second layer tended to be evenly connected to the nodes in the third layer (target interpretation), which indicates that the combinatorial pattern between the second layer and third layer are relatively independent of the methods for C-T network construction.

### 3.6. Co-Author Network and Affiliation Network

To further understand how the methodologies of THM-NP studies are employed among researchers, we constructed a co-author network and an affiliation network that were mapped with drug availability and DTI methods. The nodes in each network denote the author and affiliation, and the edges indicate that two of them appear on the same paper. The methods of DTI and drug-availability used by the corresponding author and affiliation are represented by the pie chart and the outline, respectively.

**Figure 8:**
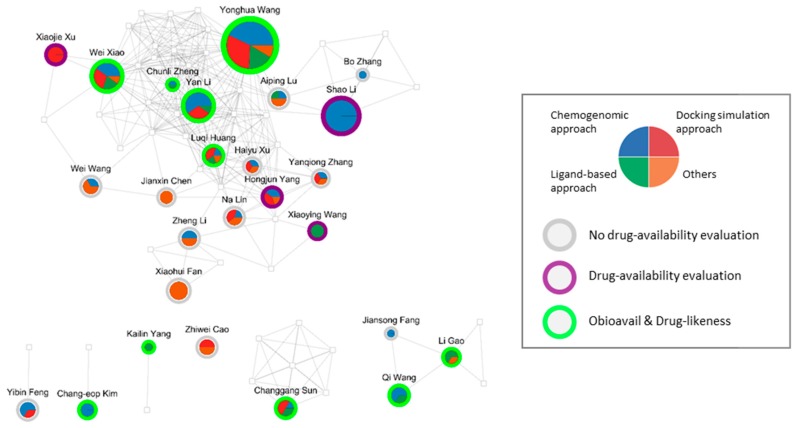
The co-author network of THM-NP studies. Circles represent corresponding authors, and squares represent non-corresponding authors. The size of the circles and squares reflect the number of occurrences in the THM-NP studies. Nodes that appeared fewer than three times were removed. The box to the right of the network represents the index for the pie chart and the outline of the circle.

In the author network, Yonghua Wang (*n* = 18) and Shao Li (*n* = 8) appeared most frequently as the corresponding author (Figure 8). More than a third of corresponding authors (52/147) combined DTI methods, such as the chemogenomic approach, docking simulation approach, and ligand-based approach. Approximately half of the corresponding authors (69/147) employed evaluation tools to screen for compounds with favorable pharmacokinetic properties, and most of them (50/69) used Obioavail and drug-likeness in combination.

We also constructed and visualized the affiliation network (Supplementary Figure S1). Northwest A&F University (*n* = 22) and China Academy of Chinese Medical Sciences (*n* = 17) appeared most frequently. Most affiliations combined various DTI methods (68/143) and employed drug-availability methods (86/143).

## 4. Discussion

In this study, we successfully identified the complex methodological trends of THM-NP research fields by analyzing the frequency of the employed methods in THM-NP studies over time and visualizing the combinatorial patterns between them. Our results showed that the number of THM-NP studies and employed databases/tools have been dramatically increased in the last decade. We also found characteristic patterns exist in combining methods of each analysis step in THM-NP studies. Finally, we showed how the diverse methods for THM-NP studies are employed and shared among researchers in the field by analyzing the co-authorship and affiliation networks.

Among the network pharmacology databases, TCMSP was the most frequently employed database for constructing herb-compound-target networks. This database was developed in 2014 and has been predominantly employed among THM-NP studies [17]. TCMSP provides a network pharmacological analysis of 499 medicinal herbs registered in the Chinese pharmacopeia along with information on ADME properties, such as bioavailability, drug-likeness, and P450, in a one-step manner. Recently, other network pharmacology databases, such as BATMAN-TCM (http://bionet.ncpsb.org/batman-tcm) and TCM-Mesh (http://mesh.tcm.microbioinformatics.org/), were developed [44,45]. They are expected to further facilitate THM-NP research fields by providing network pharmacological analysis for more than 5000 medicinal herbs.

Among the processes used in THM-NP research, the methodology for constructing a C-T network has shown the greatest change over time. In the early stages of THM-NP research, DTI methods for identifying targets of herbal ingredients relied on molecular docking simulations, which require high computational resources (Figure 5D). With the advancement of DTI prediction methods, several methodologies have been applied to THM-NP research fields that can efficiently identify the multiple targets of multiple ingredients in herbal medicines. First, the development and application of machine learning techniques and network-based methods enabled large-scale prediction of the targets of herbal medicines in terms of efficient computational costs [17,44,46,47]. Second, increased computational power made it possible to comprehensively explore potential targets of the compounds using the pharmacophore model [48]. Last, the development of databases that integrate disparate data sources provides comprehensive and high-quality information on DTIs [49]. Furthermore, recently developed DTI prediction models based on deep learning showed higher performance than other state-of-the-art models [50,51]. Such innovation in the machine learning field is expected to facilitate the development of the THM-NP research field.

To conduct THM-NP research, various tools and databases are combined in each phase of a study (Figure 7). We found that the methods for H-C network construction tend to be linked with specific methods for C-T network construction. This result indicates that there might be a preferred combinatorial pattern when choosing the methods for constructing H-C-T network. On the other hand, the combinatorial patterns between the methods for C-T network construction and target interpretation are relatively independent when compared to the previous step. This result indicates that the databases used for target interpretation tend to be chosen for the purpose of the study, while each method was preferred by different researchers in the previous steps. Further studies are needed to evaluate the reliability of network pharmacological analysis by evaluating the consistency between predicted results according to the methodologies of THM-NP studies.

There are some limitations to our study that should be noted. First, we identified potentially relevant articles using combined keywords consisting of THM-related terms and NP-related terms. Although we carefully selected search terms, we cannot guarantee that our search strategy can fully identify THM-NP studies. Second, we found potentially relevant articles only in PubMed. It is one of the largest electronic database in the world. However, there are other databases which may include other potential THM-NP studies, such as Embase, China Knowledge Resource Integrated Database (CNKI), Research Information Sharing Service (RISS), and Japan Science Technology Information Aggregator (J-stage). Last, we limited the search range of our study to English literature, which might have introduced some bias. In spite of these limitations, our results will help to improve the understanding of the methodological trends of the THM-NP research fields.

## 5. Conclusions

In conclusion, we investigated the methodological trends in THM-NP studies. Our results provide researchers with the current status of which methodologies are used in THM-NP studies and how they are applied.

## Figures and Tables

**Table 1:** The public databases related to traditional herbal medicine-network pharmacology (THM-NP) studies.

Name	Providing Information			Description	Website	PMID (Reference)
	H-C	C-T	TI			
TCMSP	○	○	○	A system of pharmacology platforms that provide information about ingredients, ADME-related properties, targets, and diseases of herbal medicines.	http://lsp.nwu.edu.cn/tcmsp.php	24735618 [26]
TCMID	○	○	○	An integrative database which stores the information of herbs, herbal compounds, targets, and their related information from different resources and through text-mining method	http://www.megabionet.org/tcmid/	23203875 [17]
TCM Databasetaiwan	○			A database that includes the information of molecular properties and substructures, TCM ingredients with their 2D and 3D structures.	http://tcm.cmu.edu.tw/	21253603 [27]
PharmMapper		○		A web server for potential drug target identification by reversed pharmacophore matching the query compound against an in-house pharmacophore model database	http://lilab.ecust.edu.cn/pharmmapper/	20430828 [28]
STITCH		○		A database that integrates disparate data sources of interactions between proteins and small molecules	http://stitch.embl.de/	18084021 [29]
TTD		○	○	A database that provides information about the therapeutic targets in the literature, targeted disease condition, and the corresponding drugs/ligands directed at each of these targets.	http://xin.cz3.nus.edu.sg/group/ttd/ttd.asp	11752352 [30]
SEA		○		A computational tool that relates proteins and chemicals based on the set-wise chemical similarity among their ligands.	http://sea.bkslab.org/	17287757 [31]
HIT		○	○	A comprehensive and fully curated database for herbal ingredients with protein target information	http://lifecenter.sgst.cn/hit/	21097881 [32]
Drugbank		○	○	A unique bioinformatics and cheminformatics resource that combines detailed drug data with comprehensive drug target information	https://www.drugbank.ca/	16381955 [33]
KEGG			○	A database resource for understanding high-level functions and utilities of the biological system from molecular-level information	https://www.genome.jp/kegg/	9847135 [34]
Gene ontology			○	The world’s largest source of information on the functions of genes	http://geneontology.org/	18792943 [35]
OMIM			○	A comprehensive and authoritative compendium of human genes and genetic phenotypes	https://www.omim.org/	11752252 [36]
PharmGkb			○	A database for the aggregation, curation, integration, and dissemination of knowledge regarding the impact of human genetic variation on drug response	https://www.pharmgkb.org/	11752281 [37]
Genecards			○	A searchable and integrated database of human genes that provides concise genomic related information, on all known and predicted human genes.	https://www.genecards.org/	12424129 [38]

H-C, herb-compound network construction; C-T, compound-target network construction; TI, target interpretation. TCMSP, Traditional Chinese Medicine Systems Pharmacology Database; TCMID, Traditional Chinese Medicine Integrated Database; STITCH, Search Tool for Interactions of Chemicals; TTD, Therapeutic Target Database; SEA, Similarity Ensemble Approach; HIT, Herb Ingredients’ Targets; KEGG, Kyoto Encyclopedia of Genes and Genomes database; OMIM, Online Mendelian Inheritance in Man; PharmGkb, The Pharmacogenetics Knowledge Base.

## Supplementary Materials

The following are available online at https://www.mdpi.com/2218-273X/9/8/362/s1. Supplementary Figure S1. The affiliation network of THM-NP studies; Supplementary Table S1. Selected THM-NP studies; Supplementary Table S2. Providing information and their types of methods employed in THM-NP studies.
