# Supplementary material for: The Methodological Trends of Traditional Herbal Medicine Employing Network Pharmacology
Source: Biomolecules. 2019 Aug 13;9(8):362. doi: 10.3390/biom9080362 (PMC6723118; doi:10.3390/biom9080362)
Supplement: Supplementary file 1 [file biomolecules-09-00362-s001.pdf]

Supplementary Documents

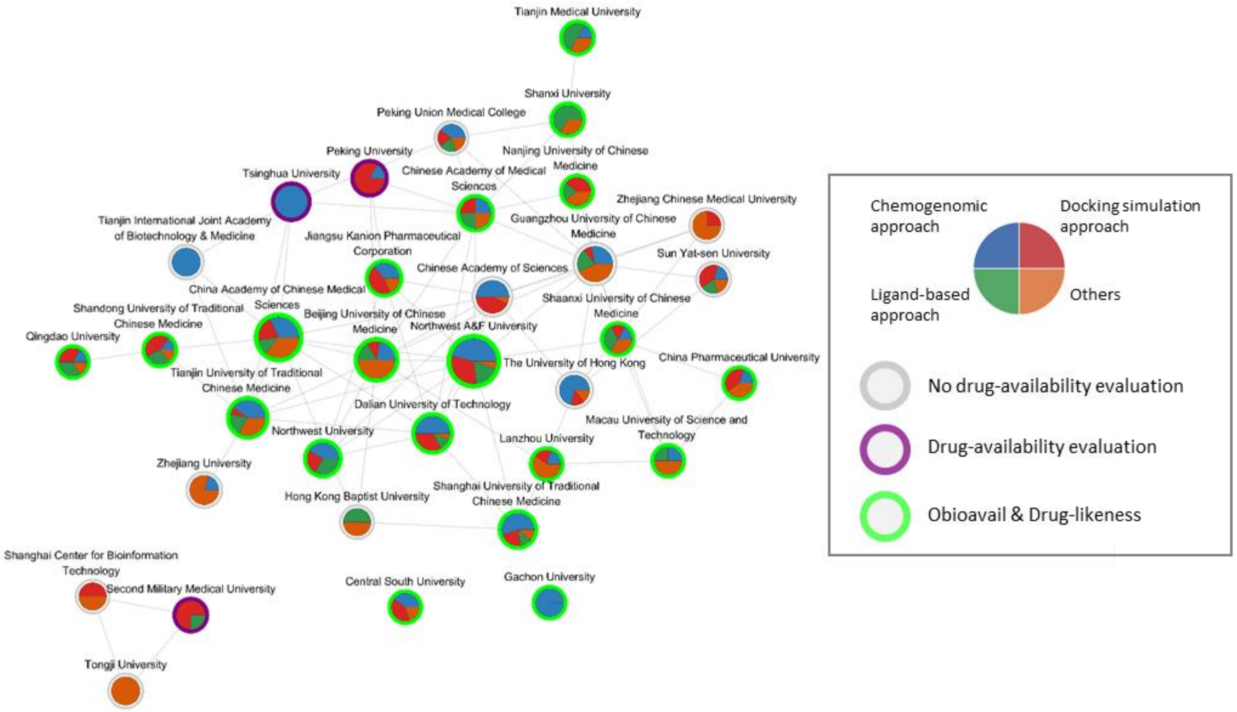

Supplementary Figure S1. The affiliation network of THM-NP studies. Circles represent the affiliations. The size of the circles and squares reflect the number of occurrences in the THM-NP studies. Nodes that appeared fewer than 3 times were removed. The box to the right of the network represents the index for the pie chart and the outline of the circle.

Supplementary Table 1. Selected THM-NP studies.

Title	Year	Author	Affiliation	The Methods for Constructing H-C Network	The Methods for Constructing C-T Network	The Methods for Target Network Interpretation	References
Drug-target network and polypharmacology studies of a Traditional Chinese Medicine for type II diabetes mellitus	2011	Jiangyong Gu, Hu Zhang, Lirong Chen, Shun Xu, Gu Yuan, Xiaojie Xu	Peking University, Zhengzhou University	Beilstein database, Chinese herbal drug database	Molecular docking	.	[1]
Potential synergistic and multitarget effect of herbal pair Chuanxiong Rhizome-Paeonia Albifora Pall on osteoarthritis disease: a computational pharmacology approach	2011	Hongzhi Ye, Chunsong Zheng, Xiaojie Xu, Mingxia Wu, Xianxiang Liu	Fujian University of Traditional Chinese Medicine, Peking University	Beilstein database, Chinese herbal drug database, Literature mining	Molecular docking	.	[2]
A System-Level Investigation into the Mechanisms of Chinese Traditional Medicine; Compound Danshen Formula for Cardiovascular Disease Treatment	2012	Xiuxiu Li, Xue Xu, Jinan Wang, Hua Yu, Xia Wang, Hongjun Yang, Haiyu Xu, Shihuan Tang, Yan Li, Ling Yang, Luqi Huang, Yonghua Wang, Shengli Yang	Chinese Academy of Medical Sciences, Northwest A&F University, Dalian University of Technology	Chinese Academy of Sciences Chemistry database, Chinese Herbal Drug Database	PharmMapper, Molecular docking	TTD, PharmGkb, Drugbank	[3]
A Systems Biology Approach to Understanding the Mechanisms of Action of Chinese Herbs for Treatment of	2012	Bohui Li, Xue Xu, Xia Wang, Hua Yu, Xiuxiu Li, Weiyang Tao, Yonghua Wang, Ling Yang	Northwest A&F University, Chinese Academy Sciences	TCMSP	TCMSP, Molecular docking	KEGG	[4]

Cardiovascular Disease							
A systems biology approach to uncovering pharmacological synergy in herbal medicines with applications to cardiovascular disease.	2012	Xia Wang, Xue Xu, Weiyang Tao, Yan Li, Yonghua Wang, Ling Yang	Northwest A&F University, Dalian University of Technology, Chinese Academy of Sciences, China Academy of Chinese Medical Sciences, Chinese Academy of Sciences	TCMSP	TCMSP	PharmGkb, TTD	[5]
Pathway-pathway network-based study of the therapeutic mechanisms by which salvianolic acid B regulates cardiovascular diseases	2012	Li Ye, Yuan He, Hao Ye, Xueping Liu, Linlin Yang, Zhiwei Cao, Kailin Tang	Shanghai Center for Bioinformation Technology, Tongji University	.	Molecular docking	.	[6]
Therapeutic effects of astragaloside IV on myocardial injuries: multi-target identification and network analysis	2012	Jing Zhao, Pengyuan Yang, Fan Li, Lin Tao, Hong Ding, Yaocheng Rui, Zhiwei Cao, Weidong Zhang	Second Military Medical University, Shanghai Center for Bioinformation Technology, Tongji University, Logistical Engineering University	.	Molecular docking	.	[7]
Understanding the molecular mechanism of interventions in treating rheumatoid arthritis patients with corresponding traditional chinese medicine patterns based on bioinformatics	2012	Miao Jiang, Cheng Lu, Gao Chen, Cheng Xiao, Qinglin Zha, Xuyan Niu, Shilin Chen, Aiping Lu	China Academy of Chinese Medical Sciences, Hubei University, China-Japan Friendship Hospital, Jiangxi University of Chinese Medicine, Hong Kong	TCMGeneDIT	TCMGeneDIT	Gene ontology	[8]

approach			Baptist University				
Deciphering the combination principles of Traditional Chinese Medicine from a systems pharmacology perspective based on Ma-huang Decoction	2013	Yao Yao, Xiaodong Zhang, Zhenzhong Wang, Chunli Zheng, Peng Li, Chao Huang, Weiyang Tao, Wei Xiao, Yonghua Wang, Luqi Huang, Ling Yang	Northwest A&F University, Zhejiang Jinju Chemcial Corporation, Jiangsu Kanion Pharmaceutical Corporation, Chinese Academy of Sciences, Northwest University	TCMSP	TCMSP, Molecular docking	TTD	[9]
Network pharmacology-based prediction of the active ingredients and potential targets of Chinese herbal Radix Curcumae formula for application to cardiovascular disease	2013	Weiyang Tao, Xue Xu, Xia Wang, Bohui Li, Yonghua Wang, Yan Li, Ling Yang	Northwest A&F University, Dalian University of Technology, Chinese Academy of Sciences	Chinese Academy of Sciences Chemistry database, Chinese Herbal Drug Database, Literature mining	TCMSP, Molecular docking	TTD, PharmGkb	[10]
Systems approaches and polypharmacology for drug discovery from herbal medicines: An example using licorice	2013	Hui Liu, Jinan Wang, Wei Zhou, Yonghua Wang, Ling Yang	Chinese Academy of Sciences, Northwest A&F University	TCMSP	TCMSP, Molecular docking	TTD, PharmGkb	[11]
Systems pharmacology uncovers Janus functions of botanical drugs; activation of host defense system and inhibition of influenza virus replication.	2013	Xia Wang, Xue Xu, Yan Li, Xiuxiu Li, Weiyang Tao, Bohui Li, Yonghua Wang, Ling Yang	Chinese Academy of Sciences, Northwest A&F University	TCMSP	TCMSP, Molecular docking	TTD, KEGG	[12]

Material basis of Chinese herbal formulas explored by combining pharmacokinetics with network pharmacology	2013	Lixia Pei, Yuanwu Bao, Sheng Liu, Jin Zheng, Xiuping Chen	Shanghai University of Traditional Chinese Medicine, University of Macau	HPLC	NCBI's entrez gene, Pubmed, CNKI	.	[13]
A network pharmacology approach to evaluating the efficacy of chinese medicine using genome-wide transcriptional expression data	2013	Leihong Wu, Yi Wang, Jing Nie, Xiaohui Fan, Yiyu Cheng	Zhejiang University, Chiatai Qingchunbao Pharmaceutical Corporation	HPLC	Microarray	KEGG	[14]
A Systems Biology-Based Investigation into the Pharmacological Mechanisms of Wu Tou Tang Acting on Rheumatoid Arthritis by Integrating Network Analysis	2013	Yanqiong Zhang, Danhua Wang, Shufang Tan, Haiyu Xu, Chunfang Liu, And Na Lin	China Academy of Chinese Medical Sciences	TCM Database@taiwan	TTD, Molecular docking	Drugbank, KEGG	[15]
An Integrative Platform of TCM Network Pharmacology and Its Application on a Herbal Formula, Qing-Luo-Yin	2013	Bo Zhang, Xu Wang, Shao Li	Tianjin International Joint Academy of Biotechnology & Medicine, Tsinghua University	HerBiomap, Literature mining	drugCIPHER	Gene ontology	[16]
Network pharmacology-based prediction of the multi-target capabilities of the compounds in Taohong Siwu decoction, and their application in osteoarthritis	2013	Chun-Song Zheng, Xiao-Jie Xu, Hong-Zhi Ye, Guang-Wen Wu, Xi-Hai Li, Hui-Feng Xu, Xian-Xiang Liu	Fujian University of Traditional Chinese Medicine, Peking University	Chinese Herbal Drug Database, Handbook of the Chemical Constutuents in Chinese Cerb	Molecular docking	TTD	[17]

				Original Plants			
Pathway Pattern-based prediction of active drug components and gene targets from H1N1 influenza's treatment with maxingshigan-yinqiaosan formula	2013	Wen Dai, Jianxin Chen, Peng Lu, Yibo Gao, Lin Chen, Xi Liu, Jianglong Song, Haiyu Xu, Di Chen, Yiping Yang, Hongjun Yang, Luqi Huang	Chinese Academy of Sciences, Beijing University of Chinese Medicine, China Academy of Chinese Medical Sciences	Literature mining	Comparative Toxicogenomics Database, Molecular docking	KEGG	[18]
The multi-target capabilities of the compounds in a TCM used to treat sepsis and their in silico pharmacology.	2013	Shitang Ma, Chengtao Feng, Xiaolin Zhang, Guoliang Dai, Changyin Li, Xiaogui Cheng, Peixun Liu, Wenzheng Ju, Hao Yu	Nanjing University of Chinese Medicine, Anhui Science and Technology University, Huainan Union University, Chinese Academy of Medical Sciences	Chinese Academy of Sciences Chemistry database, Dr. Duke's phytochemical and ethnobotanical database	Molecular docking	RCSB PDB	[19]
Towards a bioinformatics analysis of anti-Alzheimer's herbal medicines from a target network perspective	2013	Yi Sun, Ruixin Zhu, Hao Ye, Kailin Tang, Jing Zhao, Yujia Chen, Qi Liu, Zhiwei Cao	Tongji University, Shanghai Center for Bioinformation Technology	TCM-ID, Pharmacopoeia of the People's Republic of China, pubmed	HIT	HIT, KEGG	[20]
Investigation into the mechanism of Eucommia ulmoides Oliv. based on a systems pharmacology approach	2014	Yan Li, Chunxiao Han, Jinghui Wang, Wei Xiao, Zhenzhong Wang, Jingxiao Zhang, Yinfeng Yang, Shuwei Zhang, Chunzhi Ai	Dalian University of Technology, Jiangsu Kanion Pharmaceutical Corporation, Graduate School of the Chinese Academy of Sciences	TCMSP	TCMSP, Molecular docking	TTD	[21]
Systems pharmacology-based approach for	2014	Bohui Li, Weiyang Tao, Chunli Zheng, Piar	Northwest A&F University	TCMSP	TCMSP, Molecular	TTD, PharmGkb	[22]

dissecting the addition and subtraction theory of traditional Chinese medicine: An example using Xiao-Chaihu-Decoction and Da-Chaihu-Decoction		Sharali, Chao Huang, Yingxue Fu, Yonghua Wang			docking		
A network pharmacology approach to understanding the mechanisms of action of traditional medicine: Bushenhuoxue formula for treatment of chronic kidney disease	2014	Shaohua Shi, Yuepiao Cai, Xiaojun Cai, Xiaoyong Zheng, Dongsheng Cao, Faqing Ye, Zheng Xiang	Central South University, Wenzhou Medical University	Comprehensive natural products in TCM, Reaxys	Molecular docking	Drugbank, KEGG	[23]
A novel network pharmacology approachto analyse traditional herbal formulae; the Liu-Wei-Di-Huang pill as a case study	2014	Xujun Liang, Huiying Li, Shao Li	Tsinghua University	HerBiomap, TCM Database@taiwan	drugCIPHER	Gene ontology, KEGG	[24]
A systems biology-based approach to uncovering the molecular mechanisms underlying the effects of dragon's blood tablet in colitis, involving the integration of chemical analysis, ADME prediction, and network pharmacology	2014	Haiyu Xu, Yanqiong Zhang, Yun Lei, Xiumei Gao, Huaqiang Zhai, Na Lin, Shihuan Tang, Rixin Liang, Yan Ma, Defeng Li, Yi Zhang, Guangrong Zhu, Hongjun Yang, Luqi Huang	Institute of Chinese Materia Medica, China Academy of Chinese Medical Sciences, Beijing University of Chinese Medicine, Tianjin University of Traditional Chinese Medicine, Yunnan Datang Hanfang Pharmacy Corporation	TCM Database@taiwan	TTD	KEGG, Drugbank	[25]

A novel systems pharmacology model for herbal medicine injection: a case using Reduning injection.	2014	Haixing Yang, Wenjuan Zhang, Chao Huang, Wei Zhou, Yao Yao, Zhenzhong Wang, Yan Li, Wei Xiao, Yonghua Wang	Jiangsu Kanion Pharmaceutical Corporation, Northwest A & F University	.	TCMSP, SEA, STITCH	TTD, KEGG	[26]
A network pharmacology approach to determine active compounds and action mechanisms of ge-gen-qin-lian decoction for treatment of type 2 diabetes	2014	Huiying Li, Linhua Zhao, Bo Zhang, Yuyu Jiang, Xu Wang, Yun Guo, Hongxing Liu, Shao Li, Xiaolin Tong	Tsinghua University, China Academy of Chinese Medical Sciences, Tianjin International Joint Academy of Biotechnology & Medicine, Wuxi Medical School	HerBiomap	drugCIPHER	Gene ontology	[27]
A network pharmacology study of Chinese medicine QiShenYiQi to reveal its underlying multi-compound, multi-target, multi-pathway mode of action	2014	Xiang Li, Leihong Wu, Wei Liu, Yecheng Jin, Qian Chen, Linli Wang, Xiaohui Fan, Zheng Li, Yiyu Cheng	Zhejiang University, Tianjin University of Traditional Chinese Medicine	.	Microarray	.	[28]
Exploration of the mechanism of pattern-specific treatments in coronary heart disease with network pharmacology approach	2014	Hao Gu, Li Ma, Yinglong Ren, Wenjing He, Yun Wang, Yanjiang Qiao	Xinjiang Medical University, Beijing University of Chinese Medicine	Traditional Chinese Medicine Database	STITCH	Gene ontology	[29]
Identifying roles of "Jun-Chen-Zuo-Shi" component herbs of QiShenYiQi formula in treating acute	2014	Leihong Wu, Yi Wang, Zheng Li, Boli Zhang, Yiyu Cheng, Xiaohui Fan	Zhejiang University	.	Microarray	KEGG	[30]

myocardial ischemia by network pharmacology							
Network pharmacology analyses of the antithrombotic pharmacological mechanism of Fufang Xueshuantong Capsule with experimental support using disseminated intravascular coagulation rats	2014	Shujing Sheng, Jinxu Wang, Lirong Wang, Hong Liu, Peibo Li, Menghua Liu, Chaofeng Long, Chengshi Xie, Xiangqun Xie, Weiwei Su	University of Pittsburgh, Sun Yat-sen University, Guangdong Zhongsheng Pharmaceutical Corporation	HPLC	Molecular docking	Pubmed, Pubchem, Drugbank, Potential drug target database, TTD, PharmGkb	[31]
Network pharmacology study on the mechanism of traditional Chinese medicine for upper respiratory tract infection	2014	Xinzhuang Zhang, Jiangyong Gu, Liang Cao, Na Li, Yiming Ma, Zhenzhen Su, Gang Ding, Lirong Chen, Xiaojie Xu, Wei Xiao	Jiangsu Kanion Pharmaceutical Corporation, Peking University	.	Molecular docking	TTD, UTRI	[32]
TCMSP: a database of systems pharmacology for drug discovery from herbal medicines	2014	Jinlong Ru, Peng Li, Jinan Wang, Wei Zhou, Bohui Li, Chao Huang, Pidong Li, Zihu Guo, Weiyang Tao, Yinfeng Yang, Xue Xu, Yan Li, Yonghua Wang, Ling Yang	Dalian University of Technology, Chinese Academy of Sciences, Northwest A&F University	TCMSP	TCMSP	.	[33]
A network pharmacology approach to determine active ingredients and rationality of herb combinations of Modified-Simiaowan for treatment of	2015	Fangli Zhao, Li Guochun, Yanhua Yang, Le Shi, Li Xu, Lian Yin	Nanjing University of Chinese Medicine, Changzhou Seventh People's Hospital	TCMSP	Pubmed, Scifinder	KEGG, OMIM, GeneCards	[34]

gout							
A systems pharmacology approach to decipher the mechanism of danggui-shaoyao-san decoction for the treatment of neurodegenerative diseases	2015	Yunxia Luo, Qi Wang, Yongbin Zhang	Guangzhou University of Chinese Medicine	TCMSP	TCMSP	Gene ontology, KEGG	[35]
Network pharmacology-based prediction of the active ingredients and potential targets of Mahuang Fuzi Xixin decoction for application to allergic rhinitis	2015	Feng Tang, Qingfa Tang, Yuanxin Tian, Qin Fan, Yao Huang, Xiaomei Tan	Southen Medical University	TCM Database@taiwan, TCMSP	TCMSP, Molecular docking	TTD, PharmGkb	[36]
Systems Pharmacology Dissecting Holistic Medicine for Treatment of Complex Diseases: An Example Using Cardiocerebrovascular Diseases Treated by TCM	2015	Yonghua Wang, Chunli Zheng, Chao Huang, Yan Li, Xuetong Chen, Ziyin Wu, Zhenzhong Wang, Wei Xiao, Boli Zhang	Tianjin University of Traditional Chinese Medicine, Northwest A&F University, Dalian University of Technology, State Key Laboratory of New-Tech for Chinese Medicine Pharmaceutical Process	Literature mining	TCMSP, Molecular docking, Drugbank	KEGG	[37]
Systems pharmacology-based dissection of mechanisms of Chinese medicinal formula Bufei Yishen as an effective treatment for chronic	2015	Jiansheng Li, Peng Zhao, Ya Li, Yange Tian, Yonghua Wang	Henan University of Chinese Medicine, Northwest A&F University	Chinese Academy of Sciences Chemistry database, TCMSP,	TCMSP, Molecular docking	TTD, PharmGkb	[38]

obstructive pulmonary disease				Literature mining			
Anti-osteoporosis effect of Epimedium via an estrogen-like mechanism based on a system-level approach.	2015	Feifei Xu, Yan Ding, Yingying Guo, Baoyue Liu, Zinong Kou, Wei Xiao, Jingbo Zhu	Dalian Polytechnic University, Jiangsu Kanion Pharmaceutical Corporation	TCMSP	TCMSP, SEA, STITCH, Molecular docking, TTD	KEGG	[39]
Bioactivity-integrated UPLC/Q-TOF-MS of Danhong injection to identify NF-kappaB inhibitors and anti-inflammatory targets based on endothelial cell culture and network pharmacology	2015	Xiaoqing Jiang, Bin Lv, Pan Li, Xianghui Ma, Ting Wang, Qian Zhou, Xiaoying Wang, Xiumei Gao	Tianjin University of Traditional Chinese Medicine	UPLC	PharmMapper	Molecule Annotation System	[40]
The study on the material basis and the mechanism for anti-renal interstitial fibrosis efficacy of rhubarb through integration of metabonomics and network pharmacology	2015	Zheng Xiang, Hao Sun, Xiaojun Cai, Dahui Chen, Xiaoyong Zheng	Wenzhou Medical University	Comprehensive natural products in TCM, Reaxys	Molecular docking	TTD, Drugbank, OMIM, Genetic Association database	[41]
Deciphering the underlying mechanisms of Diesun Miaofang in traumatic injury from a systems pharmacology perspective.	2015	ChunSong Zheng, ChangLong Fu, CaiBin Pan, HongJuan Bao, XingQiang Chen, HongZhi Ye, JinXia Ye, GuangWen Wu, XiHai Li, HuiFeng Xu, XiaoJie Xu, XianXiang Liu	Fujian University of Traditional Chinese Medicine, Xiamen Medical College, Peking University	Chinese Herbal Drug Database	Molecular docking	.	[42]

A Systems Biology Perspective on the Molecular Mechanisms Underlying the Therapeutic Effects of Buyang Huanwu Decoction on Ischemic Stroke	2015	Qiuyan Guo, Micun Zhong, Haiyu Xu, Xia Mao, Yanqiong Zhang, Na Lin	China Academy of Chinese Medical Sciences	TCM Database@taiwan	TTD, Molecular docking	Gene ontology, KEGG	[43]
A Systems Biology-Based Investigation into the Pharmacological Mechanisms of Sheng-ma-bie-jia-tang Acting on Systemic Lupus Erythematosus by Multi-Level Data Integration	2015	Lin Huang, Qi Lv, Fenfen Liu, Tieliu Shi, Chengping Wen	East China Normal University, Zhejiang Chinese Medical University, Guangxi Medical University	TCMID	STITCH, TCMID	KEGG	[44]
Herb Network Analysis for a Famous TCM Doctor's Prescriptions on Treatment of Rheumatoid Arthritis	2015	Yan Li, Rui Li, Zibo Ouyang, Shao Li	Wannan Medical College, Tsinghua University	HerBiomap	drugCIPHER	KEGG	[45]
Network pharmacology-based antioxidant effect study of zhi-zi-da-huang decoction for alcoholic liver disease	2015	Li An, Fang Feng	China Pharmaceutical University	HPLC	Molecular docking	.	[46]
Tetramethylpyrazine identified by a network pharmacology approach ameliorates methotrexate-induced oxidative organ injury	2015	Bo Zhang, Cheng Lu, Ming Bai, Xiaojuan He, Yong Tan, Yanqin Bian, Cheng Xiao, Ge Zhang, Aiping Lu, Shao Li	China Academy of Chinese Medical Sciences, Tsinghua University, China-Japan Friendship Hospital, Tianjin	.	drugCIPHER	KEGG	[47]

			International Joint Academy of Biotechnology & Medicine				
Molecular targets of Chinese herbs: a clinical study of hepatoma based on network pharmacology	2016	Li Gao, Xiaodong Wang, Yangyang Niu, Dandan Duan, Xue Yang, Jian Hao, Cuihong Zhu, Dan Chen, Kexin Wang, Xuemei Qin, Xiongzhi Wu	Shanxi University, Tianjin Medical University, Tianjin People's Hospital	TCMSP, TCMID, TCM-PTD	ChemMapper	HIT	[48]
Systematic understanding the mechanisms of vitiligo pathogenesis and its treatment by Qubaibabuqi formula	2016	Tianli Pei, Chunli Zheng, Chao Huang, Xuetong Chen, Zihu Guo, Yingxue Fu, Jianling Liu, Yonghua Wang	Northwest A&F University	TCMSP	WES	Gene ontology, KEGG	[49]
Systems Pharmacology Dissection of the Integrated Treatment for Cardiovascular and Gastrointestinal Disorders by Traditional Chinese Medicine	2016	Wenjuan Zhang, Qin Tao, Zihu Guo, Yingxue Fu, Xuetong Chen, Piar Sharali, Mohamed Shahen, Jinglin Zhu, Jun Xue, Yaofei Bai, Ziyin Wu, Zhenzhong Wang, Wei Xiao, Yonghua Wang	Chinese Medicine Pharmaceutical Process, Northwest A&F University, Northwest University	TCMSP	TCMSP, Molecular docking	Gene ontology, KEGG	[50]
Systems pharmacology exploration of botanic drug pairs reveals the mechanism for treating different diseases	2016	Wei Zhou, Jinan Wang, Ziyin Wu, Chao Huang, Aiping Lu, Yonghua Wang	Shihezi University, Northwest A&F University, Shanghai Academy of Chinese Medical Sciences, China Academy of	TCMSP	HIT, TTD, SEA, STITCH, TCMSP	HIT, TTD, Drugbank	[51]

			Chinese Medical Sciences				
Systems Pharmacology Uncovers the Multiple Mechanisms of Xijiao Dihuang Decoction for the Treatment of Viral Hemorrhagic Fever	2016	Jianling Liu, Tianli Pei, Jiexin Mu, Chunli Zheng, Xuetong Chen, Chao Huang, Yingxue Fu, Zongsuo Liang, Yonghua Wang	Zhejiang Sci-Tech University, Northwest University, Northwest A&F University	TCMSP	TCMSP, Molecular docking	Gene ontology, KEGG	[52]
Systems-Pharmacology Dissection of Traditional Chinese Medicine Compound Saffron Formula Reveals Multi-scale Treatment Strategy for Cardiovascular Diseases	2016	Jianling Liu, Jiexin Mu, Chunli Zheng, Xuetong Chen, Zihu Guo, Chao Huang, Yingxue Fu, Guihua Tian, Hongcai Shang, Yonghua Wang	Northwest University, Northwest A&F University, Beijing University of Chinese Medicine	TCMSP	WES	Gene ontology, KEGG	[53]
Network pharmacology dissection of multiscale mechanisms of herbal medicines in stage IV gastric adenocarcinoma treatment.	2016	Li Gao, Jian Hao, YangYang Niu, Miao Tian, Xue Yang, CuiHong Zhu, XiuLi Ding, XiaoHui Liu, HaoRan Zhang, Chang Liu, XueMei Qin, XiongZhi Wu	Shanxi University, Tianjin Medical University	TCMSP	ChemMapper, HIT	.	[54]
Systems Pharmacology Based Study of the Molecular Mechanism of SiNiSan Formula for Application in Nervous and Mental Diseases.	2016	Xia Shen, Zhenyu Zhao, Xuan Luo, Hao Wang, Benxiang Hu, Zihu Guo	Shaanxi University of Chinese Medicine, Northwest A&F University	TCMSP, Literature mining	WES	PharmGkb	[55]
Drug discovery of neurodegenerative disease	2016	Zhipeng Ke, Xinzhuang Zhang,	Peking University, Jiangsu Kanion	TCMN	Molecular docking	TTD, Drugbank	[56]

through network pharmacology approach in herbs		Zeyu Cao, Yue Ding, Na Li, Liang Cao, Tuanjie Wang, Chenfeng Zhang, Gang Ding, Zhenzhong Wang, Xiaojie Xu, Wei Xiao	Pharmaceutical Corporation				
A novel strategy of profiling the mechanism of herbal medicines by combining network pharmacology with plasma concentration determination and affinity constant measurement.	2016	Langdong Chen, Diya Lv, Dongyao Wang, Xiaofei Chen, Zhenyu Zhu, Yan Cao, Yifeng Chai	Second Military Medical University	Chemistry database of Shanghai Institute of Organic Chemistry, Chinese Academy of Science	Molecular docking	.	[57]
A network analysis of the Chinese medicine Lianhua-Qingwen formula to identify its main effective components	2016	Chunhua Wang, Yi Zhong, Yan Zhang, Jinping Liu, Yuefei Wang, Weina Jia, Guocai Wang, Zheng Li, Yan Zhu, Xiumei Gao	Tianjin University of Traditional Chinese Medicine, Zhejiang University, Jinan University of Traditional Chinese Medicine	UPLC	TCMSP, STITCH	OMIM	[58]
A Network Pharmacology Approach to Explore the Pharmacological Mechanism of Xiaoyao Powder on Anovulatory Infertility	2016	Huiping Liu, Liuting Zeng, Kailin Yang, Guomin Zhang	Hunan University of Chinese Medicine	TCM Database@taiwan, TCMSP	PharmMapper	TTD, KEGG	[59]
BATMAN-TCM: a Bioinformatics Analysis Tool for Molecular	2016	Zhongyang Liu, Feifei Guo, Yong Wang, Chun Li, Xinlei Zhang,	Beijing Institute of Radiation Medicine, National Center for	TCMID	BATMAN-TCM, Molecular	Gene ontology, KEGG, OMIM, TTD	[60]

mechANism of Traditional Chinese Medicine		Honglei Li, Lihong Diao, Jiangyong Gu, Wei Wang, Dong Li, Fuchu He	Protein Sciences Beijing, Chinese Academy of Medical Sciences, Beijing University of Chinese Medicine, Beijing Genestone Technology, Peking University		docking		
Bioinformatics investigation of therapeutic mechanisms of Xuesaitong capsule treating ischemic cerebrovascular rat model with comparative transcriptome analysis	2016	Jiangquan Liao, Benjun Wei, Hengwen Chen, Yongmei Liu, Jie Wang	China Academy of Chinese Medical Sciences, Beijing University of Chinese Medicine, Hubei University of Chinese Medicine	.	Microarray	Gene ontology, KEGG	[61]
Herb-target interaction network analysis helps to disclose molecular mechanism of traditional Chinese medicine	2016	Hao Liang, Hao Ruan, Qi Ouyang, Luhua Lai	Peking University	Pharmacopoeia of the People's Republic of China	Molecular docking	.	[62]
Identification of the anti-tumor activity and mechanisms of nuciferine through a network pharmacology approach	2016	Quan Qi, Rui Li, Huiying Li, Yubing Cao, Ming Bai, Xiaojing Fan, Shuyan Wang, Bo Zhang, Shao Li	Tianjin University of Traditional Chinese Medicine, Tsinghua University, Tianjin International Joint Academy of Biotechnology & Medicine	drugCHIPER-CS	drugCIPHER	.	[63]
Study on material base and action mechanism of compound Danshen	2016	Wei Zhou, Wenfeng Yuan, Chao Chen, Shumei Wang,	Guangdong Pharmaceutical University	TCMSP	Tanimoto coefficient	Gene ontology, KEGG	[64]

dripping pills for treatment of atherosclerosis based on modularity analysis		Shengwang Liang					
To Unveil the Molecular Mechanisms of Qi and Blood through Systems Biology-Based Investigation into Si-Jun-Zi-Tang and Si-Wu-Tang formulae	2016	Jing Sun, Li Zhang, Yujun He, Kun Zhang, Liping Wu, Yongsheng Fan, Zhijun Xie	Zhejiang Chinese Medical University, Hangzhou Traditional Chinese Medical Hospital	TCMID	STITCH	Gene ontology, KEGG	[65]
Identification of "Multiple Components-Multiple Targets-Multiple Pathways" Associated with Naoxintong Capsule in the Treatment of Heart Diseases Using UPLC/Q-TOF-MS and Network Pharmacology.	2016	Xianghui Ma, Bin Lv, Pan Li, Xiaoqing Jiang, Qian Zhou, Xiaoying Wang, Xiumei Gao	Tianjin University of Traditional Chinese Medicine	UPLC	PharmMapper	Bio database	[66]
Systems-biology dissection of mechanisms and chemical basis of herbal formula in treating chronic myocardial ischemia.	2016	Shuzhen Guo, Peng Li, Bangze Fu, Wenjing Chuo, Kuo Gao, Wuxia Zhang, Junyao Wang, Jianxin Chen, Wei Wang	Beijing University of Chinese Medicine, Shanxi Agricultural University	TCMSP, Literature mining	Microarray	Drugbank, TTD, FDA orange book, Gene ontology, KEGG	[67]
Genomic expression profiling and bioinformatics analysis on diabetic nephrology with ginsenoside Rg3.	2016	Juan Wang, Chunli Cui, Li Fu, Zili Xiao, Nanzi Xie, Yang Liu, Lu Yu, Haifeng Wang, Bangzhen Luo	Tongji University, Shanghai Jinfang Biotechnology Corporation, Dalian Fusheng Natural Pharmaceutical	.	RNA-seq	Gene ontology, KEGG	[68]

			Development Corporation				
Systems Pharmacology Dissection of Multiscale Mechanisms of Action for Herbal Medicines in Treating Rheumatoid Arthritis	2017	Jinghui Wang, Yan Li, Yinfeng Yang, Jian Du, Miaoqing Zhao, Feng Lin, Shuwei Zhang, Bin Wang	Dalian University of Technology, Dalian Ocean University	TCMSP	TCMSP, Molecular docking	KEGG	[69]
Uncovering the Pharmacological Mechanism of Astragalus Salvia Compound on Pregnancy-Induced Hypertension Syndrome by a Network Pharmacology Approach	2017	Liuting Zeng, Kailin Yang, Jinwen Ge	Hunan University of Chinese Medicine	TCM Database@taiwan, TCMSP	PharmMapper	Gene ontology	[70]
A network pharmacology approach to establish the pharmacological mechanism of JiaWeiXianJiTang on inflammatory bowel disease	2017	Lingjia Tao, Wenting Chen, Lin Jing, Qing Ji, Jianlin Ren	Shanghai University of Traditional Chinese Medicine	TCM-PTD	TCM-PTD	Wangfang Database, CNKI, VIP database	[71]
Network pharmacology-based approach of novel traditional Chinese medicine formula for treatment of acute skin inflammation in silico	2017	Hsinchieh Tang, Hungjin Huang, Chengchun Lee, Calvin Yuchian Chen	China Medical University Hospital, China Medical University	.	Molecular docking	.	[72]
Network pharmacology-	2017	Guohua Yu, Yanqiong	China Academy of	Chinese	Medchem,	OMIM, KEGG	[73]

based identification of key pharmacological pathways of Yin-Huang-Qing-Fei capsule acting on chronic bronchitis		Zhang, Weiqiong Ren, Ling Dong, Junfang Li, Ya Geng, Yi Zhang, Defeng Li, Haiyu Xu, Hongjun Yang	Chinese Medical Sciences	Academy of Sciences Chemistry database	Drugbank		
PDTCM: a systems pharmacology platform of traditional Chinese medicine for psoriasis.	2017	Dongmei Wang, Jiangyong Gu, Wei Zhu, Fang Luo, Lirong Chen, Xiaojie Xu, Chuanjian Lu	Guangdong Provincial Academy of Chinese Medical Sciences	Reaxys, Chinese Natural Product Database, Traditional Chinese Medicine Database, Chinese Herbal Drug Database	Molecular docking	OMIM, KEGG	[74]
Predicted molecular targets and pathways for germacrone, curdione, and furanodiene in the treatment of breast cancer using a bioinformatics approach	2017	Qi Kong, Yong Ma, Jie Yu, Xiuping Chen	Peking Union Medical College, Shanxian Central Hospital, University of Macau	.	BATMAN-TCM, Molecular docking	Gene ontology, KEGG	[75]
The Combination of Three Components Derived from Sheng MaiSan Protects Myocardial Ischemic Diseases and Inhibits Oxidative Stress via Modulating MAPKs and JAK2-STAT3 Signaling Pathways Based on Bioinformatics Approach	2017	Fang Li, Yu Zhang, Donglin Zeng, Yu Xia, Xiaoxue Fan, Yisha Tan, Junping Kou, Boyang Yu	China Pharmaceutical University	.	PharmMapper, Literature mining	Gene ontology, KEGG	[76]

A Network Pharmacology-Based Study on the Hepatoprotective Effect of Fructus Schisandrae.	2017	ing Hong, Yongsheng Zhang, Sha Li, Hor TanYue, Ning Wang, Shuzhen Mu, Xiaojiang Hao, Yibin Feng	The University of Hong Kong, Guangzhou University of Chinese Medicine, Zhejiang Chinese Medical University, Chinese Academy of Sciences	.	Molecular docking	.	[77]
A Biomedical Investigation of the Hepatoprotective Effect of Radix salviae miltiorrhizae and Network Pharmacology-Based Prediction of the Active Compounds and Molecular Targets	2017	Ming Hong, Sha Li, Ning Wang, Horyue Tan, Fan Cheung, Yibin Feng	The University of Hong Kong	TCMSP, TCM Database@taiwan	TCMSP	.	[78]
A Methodology for Cancer Therapeutics by Systems Pharmacology-Based Analysis: A Case Study on Breast Cancer-Related Traditional Chinese Medicines	2017	Yan Li, Jinghui Wang, Feng Lin, Yinfeng Yang, Sushing Chen	University of Florida, Dalian University of Technology	Chinese Academy of Sciences Chemistry database, Chinese Herbal Drug Database	TCMSP	KEGG, Gene ontology	[79]
Exploring the pharmacological mechanism of Yanghe Decoction on HER2-positive breast cancer by a network pharmacology approach	2017	Liuting Zeng, Kailin Yang	Hunan University of Chinese Medicine	TCM Database@taiwan, TCMSP	PharmMapper	Gene ontology, KEGG	[80]

Identification of the active compounds and significant pathways of yinchenhao decoction based on network pharmacology	2017	Jihan Huang, Fan Cheung, Horyue Tan, Ming Hong, Ning Wang, Juan Yang, Yibin Feng, Qingshan Zheng	Shanghai University of Traditional Chinese Medicine, The University of Hong Kong	TCMSP	TCMSP	Gene ontology, KEGG, OMIM	[81]
Molecular Targets and Associated Potential Pathways of Danlu Capsules in Hyperplasia of Mammary Glands Based on Systems Pharmacology	2017	Jihan Huang, Haitao Tang, Sumin Cao, Yingchun He, Yibin Feng, Kun Wang, Qingshan Zheng	Shanghai University of Traditional Chinese Medicine, The University of Hong Kong, Suzhong Pharmaceutical Group Corporation	TCMSP	TCMSP	Gene ontology, KEGG, OMIM	[82]
Systems-level mechanisms of action of Panax ginseng: a network pharmacological approach	2017	Sayoon Park, Jihun Park, Hyosu Kim, Choongyeol Lee, Hae-jeung Lee, Kisung Kang, Chang-eop Kim	Gachon University	TCMSP	TCMSP	PANTHER, OMIM, KEGG	[83]
Network pharmacology-based and clinically relevant prediction of the active ingredients and potential targets of Chinese herbs in metastatic breast cancer patients.	2017	Yu Mao, Jian Hao, ZiQi Jin, YangYang Niu, Xue Yang, Dan Liu, Rui Cao, XiongZhi Wu	Tianjin Medical University	TCMSP, TCMID	ChemMapper, HIT	KEGG, TTD	[84]
Network-pharmacology-based validation of TAMS/CXCL-1 as key mediator of XIAOPI formula preventing breast cancer development and metastasis.	2017	Neng Wang, Yifeng Zheng, Jiangyong Gu, Youli Cai, Shengqi Wang, Fengxue Zhang, Jianping Chen, Honglin Situ, Yi Lin, Zhiyu Wang	Guangzhou University of Chinese Medicine, The University of Hong Kong	TCMSP, TCMID, BATMAN-TCM	TCMSP, TCMID, BATMAN-TCM	GeneCards, KEGG, Gene ontology	[85]

A Network Pharmacology Approach to Determine the Active Components and Potential Targets of Curculigo Orchioides in the Treatment of Osteoporosis.	2017	Nani Wang, Guizhi Zhao, Yang Zhang, Xuping Wang, Lisha Zhao, Pingcui Xu, Dan Shou	Zhejiang Academy of Traditional Chinese Medicine, Second Military Medical University	UPLC, TCMSP	PharmMapper	KEGG	[86]
Network pharmacology-based study on the mechanism of action for herbal medicines in Alzheimer treatment	2017	Jiansong Fang, Ling Wang, Tian Wu, Cong Yang, Li Gao, Haobin Cai, Junhui Liu, Shuhuan Fang, Yunbo Chen, Wen Tan, Qi Wang	Guangzhou University of Chinese Medicine, South China University of Technology, Shanxi University, Guangxi University of Chinese Medicine, Guangdong University of Technology	TCMSP, TCMID, TimTec plant extracts database	AlzhCPI	KEGG	[87]
A network pharmacology approach to investigate the pharmacological effects of Guizhi Fuling Wan on uterine fibroids	2017	Liuting Zeng, Kailin Yang, Huiping Liu, Guomin Zhang	Hunan University of Chinese Medicine	SciFinder	PharmMapper	KEGG, Gene ontology, OMIM, GeneCards	[88]
A network pharmacology-based strategy deciphers the underlying molecular mechanisms of Qixuehe Capsule in the treatment of menstrual disorders	2017	Yanqiong Zhang, Xia Mao, Jing Su, Ya Geng, Rui Guo, Shihuan Tang, Junfang Li, Xuefeng Xiao, Haiyu Xu, Hongjun Yang	China Academy of Chinese Medical Sciences, Shandong University of Traditional Chinese Medicine, Tianjin University of Traditional Chinese Medicine	Organchem	Metadrug	Drugbank	[89]
Combining Chemical Profiling and Network	2017	Tongchuan Suo, Jinping Liu, Xi Chen,	Tianjin University of Traditional Chinese	UPLC	TCMSP, STITCH	KEGG	[90]

Analysis to Investigate the Pharmacology of Complex Prescriptions in Traditional Chinese Medicine		Hua Yu, Tenglong Wang, Congcong Li, Yuefei Wang, Chunhua Wang, Zheng Li	Medicine				
Design of new traditional Chinese medicine herbal formulae for treatment of type 2 diabetes mellitus based on network pharmacology.	2017	Ruifeng Hu, Xiaobo Sun	Chinese Academy of Medical Sciences	Medicinal Plants Database	TarNet	T2D-Db, T-HOD, T2D@ZJU	[91]
Dissecting the Underlying Pharmaceutical Mechanism of Chinese Traditional Medicine Yun-Pi-Yi-Shen-Tong-Du-Tang Acting on Ankylosing Spondylitis through Systems Biology Approaches	2017	Duoli Xie, Lin Huang, Guanghui Zhao, Yiran Yu, Jiawei Gao, Haichang Li, Chengping Wen	Zhejiang Chinese Medical University, Guangzhou University of Chinese Medicine	TCMID	TCMID, STITCH	Gene ontology, KEGG	[92]
Molecular mechanisms of the analgesic action of Wu-tou Decoction on neuropathic pain in mice revealed using microarray and network analysis	2017	Yanqiong Zhang, Chao Wang, Qiuyan Guo, Chunyan Zhu, Chen Yan, Danni Sun, Qionghong Xu, Na Lin	China Academy of Chinese Medical Sciences	.	Microarray	KEGG	[93]
Network pharmacology exploration reveals endothelial inflammation as a common mechanism for stroke and coronary artery disease treatment of	2017	Ming Lyu, Chunlin Yan, Haixin Liu, Taiyi Wang, Xinhui Shi, Jinping Liu, John Orgah, Guanwei Fan, Jihong Han, Xiaoying	Tianjin University of Traditional Chinese Medicine, Nankai University, Harvard Medical School	TCMSP, TCM Database@taiwan, TCM-ID, TCMGeneDIT, Literature mining	TCMSP, TCMID	IPA, OMIM, CADGene, NCBI Gene database, GeneCards, MalaCards	[94]

Danhong injection		Wang, Yan Zhu					
Systems Pharmacology-based strategy to screen new adjuvant for hepatitis B vaccine from Traditional Chinese Medicine Ophiocordyceps sinensis	2017	Jingbo Wang, Rui Liu, Baoxiu Liu, Yan Yang, Jun Xie, Naishuo Zhu	Fudan University	TCMSP	TCMSP	Gene ontology, KEGG	[95]
Deciphering Key Pharmacological Pathways of Qingdai Acting on Chronic Myeloid Leukemia Using a Network Pharmacology-Based Strategy	2018	Huayao Li, Lijuan Liu, Cun Liu, Jing Zhuang, Chao Zhou, Jing Yang, Chundi Gao, Gongxi Liu, Qingliang Lv, Changgang Sun,	Shandong University of Traditional Chinese Medicine, Affiliated Hospital of Weifang Medical University, Weifang People's Hospital	TCMSP	SwissTargetPrediction, Molecular docking	Pubmed, Drugbank, OMIM, Gene ontology, KEGG, TTD	[96]
Exploring Pharmacological Mechanisms of Xuefu Zhuyu Decoction in the Treatment of Traumatic Brain Injury via a Network Pharmacology Approach	2018	Zhiping Miao, Chu Chen, Mohamed Shahen, Siddiq Rahmanur, Alagawany, Mahmoud, Mohamed E Abd Elhack, Heping Zhao, Airong Qian	Central South University, Xinjiang Medical University, Xiangya Hospital	TCMSP	TCMSP, Molecular docking	TTD, OMIM, Gene ontology, KEGG	[97]
Exploring the Mechanism of Danshen against Myelofibrosis by Network Pharmacology and Molecular Docking	2018	Jie Li, Xiaoran Ma, Cun Liu, Huayao Li, Jing Zhuang, Chundi Gao, Chao Zhou, Lijuan Liu, Kejia Wang, Changgang Sun	Shandong University of Traditional Chinese Medicine, Weifang Traditional Chinese Hospital, Qingdao University	TCMSP	SwissTargetPrediction, TCMSP, Molecular docking	OMIM, TTD, Gene ontology, KEGG	[98]
Integrated Metabolomics and Network Pharmacology Approach to	2018	Hanqing Pang, Shijun Yue, Yuping Tang, Yanyan Chen, Yajie	Shaanxi University of Chinese Medicine, Nanjing University of	TCMSP	PharmMapper, STITCH, SEA, Drugbank,	KEGG	[99]

Explain Possible Action Mechanisms of Xin-Sheng-Hua Granule for Treating Anemia		Tan, Yujie Cao, Xuqin Shi, Guisheng Zhou, An Kang, Shengliang Huang, Yajun Shi, Jing Sun, Zhishu Tang, Jinao Duan	Chinese Medicine, Jiangsu Revolence Pharmaceutical Corporation		TTD, HIT, Literature mining		
Investigate the mechanisms of Chinese medicine Fuzhengkangai towards EGFR mutation-positive lung adenocarcinomas by network pharmacology.	2018	Zhitong Bing, Zhiyuan Cheng, Danfeng Shi, Xinkui Liu, Jinhui Tian, Xiaojun Yao, Jingyun Zhang, Yongfeng Wang, Kehu Yang	School of Basic Medical Science of Lanzhou University	TCMSP	Microarray, Molecular docking	KEGG	[100]
Systems Pharmacology Dissection of Multi-Scale Mechanisms of Action of Huo-Xiang-Zheng-Qi Formula for the Treatment of Gastrointestinal Diseases.	2018	Miaoqing Zhao, Yangyang Chen, Chao Wang, Wei Xiao, Shusheng Chen, Shuwei Zhang, Ling Yang, Yan Li	Dalian University of Technology, Northwest A&F University, University of Florida, Shanghai University of Traditional Chinese Medicine	TCMSP, Chinese Academy of Sciences Chemistry, HIT, TCM Database@taiwan, TCMID	TCMSP, Molecular docking	TTD, DrugBank, PharmGkb, Gene ontology	[101]
A Network Pharmacology-Based Approach to Investigate the Novel TCM Formula against Huntington's Disease and Validated by Support Vector Machine Model	2018	Wenjie Dai, Hsinyi Chen, Calvin Yuchian Chen	Sun Yat-sen University	TCM Database@taiwan	QSAR, Molecular docking	KEGG	[102]
A Systems Biology-Based Approach to Uncovering Molecular Mechanisms Underlying Effects of Traditional Chinese	2018	Chao Zhou, Lijuan Liu, Jing Zhuang, Junyu Wei, Tingting Zhang, Chundi Gao, Cun Liu, Huayao Li, Hongzong	WeiFang Traditional Chinese Hospital, Shandong University of Traditional Chinese Medicine, Qingdao	CHDD	Molecular docking	OMIM	[103]

Medicine Qingdai in Chronic Myelogenous Leukemia,		Si, Changgang Sun	University, Affiliated Hospital of Weifang Medical University				
A systems pharmacology-oriented discovery of a new therapeutic use of the TCM formula Liuweiwuling for liver failure	2018	Jiabo Wang, Herong Cui, Ruilin Wang, Congen Zhang, Ming Niu, Zhaofang Bai, Genhua Xu, Pengyan Li, Wenyan Jiang, Jingjing Han, Xiao Ma, Guangming Cai, Ruisheng Li, Liping Zhang, Xiaohe Xiao	302 Military Hospital of China, Beijing University of Traditional Chinese Medicine, Chengdu University of Traditional Chinese Medicine	TCMSP, TCM Database@Taiwan	PharmMapper	OMIM, Gene ontology, KEGG	[104]
Network pharmacology-based strategy for predicting active ingredients and potential targets of Yangxinshi tablet for treating heart failure	2018	Langdong Chen, Yan Cao, Hai Zhang, Diya Lv, Yahong Zhao, Yanjun Liu, Guan Ye, Yifeng Chai	Second Military Medical University, Tongji University School of Medicine, Shanghai Pharmaceuticals Holding Corporation	In-house database	Molecular docking	Drugbank, PharmGkb, Potential Drug Target Database, TTD, KEGG, Gene ontology	[105]
Network Pharmacology Uncovers Anticancer Activity of Mammea-Type Coumarins from Calophyllum brasiliense	2018	JuanCarlos Gómez-Verjan, NadiaAlejandra Rivero-Segura, Edgar Estrella-Parra, Ruth Rincón-Heredia, Abraham Madariaga-Mazón, Edgar Flores-Soto, Mario González-Meljem, Marco Cerbón, Ricardo Reyes-Chilpa	Instituto Nacional de Geriatría, Universidad Nacional Autónoma de México	NMR	DAAR-CPI, SEA, SwissTarget, STITCH	.	[106]

A Systems-Level Analysis of Mechanisms of Platycodon grandiflorum Based on A Network Pharmacological Approach	2018	Musun Park, Sayoon Park, Hae-jeung Lee, Chang-eop Kim	Gachon University	TCMSP	TCMSP	PANTHER, KEGG, OMIM	[107]
Network pharmacology-based prediction of active compounds and molecular targets in Yijin-Tang acting on hyperlipidaemia and atherosclerosis	2018	Ayeong Lee, Won Park, Taewook Kang, Minho Cha, Jinmi Chun	Korea Institute of Oriental Medicine, Insilicogen Corporation, Sookmyung Women's University	Korean traditional knowledge portal database	STITCH	KEGG	[108]
Protective Effect of Artemisia argyi and Its Flavonoid Constituents against Contrast-Induced Cytotoxicity by Iodixanol in LLC-PK1 Cells	2018	Dahae Lee, Chang-eop Kim, Sayoon Park, Kemok Kim, Nguyentuan Hiep, Dongho Lee, Hyukjai Jang, Jaewook Lee, Kisung Kang	Sungkyunkwan University, Gachon University, Korea University, University of Ulsan College of Medicine, Korea Institute of Science and Technology	.	TCMSP	PharmGkb, TTD	[109]
Systematic Investigation of Scutellariae Barbatae Herba for Treating Hepatocellular Carcinoma Based on Network Pharmacology	2018	Benjiao Gong	Qingdao University, Yantai Hospital of Traditional Chinese Medicine,	TCMSP	PubChem, PharmMapper	OncoDB.HCC, Liverome, Gene ontology, KEGG	[110]
Systematic Understanding of the Mechanism of Baicalin against Ischemic Stroke through a Network Pharmacology Approach	2018	Tian Xu, Chongyang Ma, Shuning Fan, Nang Deng, Yajun Lian, Ling Tan, Weizhe Du, Shuang Zhang, Shuling Liu, Beida Ren, Zhenhan Li, Qinguo	Beijing University of Chinese Medicine	.	Pubmed, PharmMapper	OMIM, GAD, TTD, Gene ontology, KEGG	[111]

		Wang, Xueqian Wang					
Systems pharmacology analysis of synergy of TCM; an example using saffron formula	2018	Jianling Liu, Jingjing Liu, Fengxia Shen, Zonghui Qin, Meng Jiang, Jinglin Zhu, Zhenzhong Wang, Jun Zhou, Yingxue Fu, Xuetong Chen, Chao Huang, Wei Xiao, Chunli Zheng, Yonghua Wang	Northwest A&F University, Northwest University, Jiangsu Kanion Parmaceutical Corporation	TCMSP	TCMSP, WES	PharmGkb, TTD, Gene ontology, KEGG	[112]
Systems pharmacology approach to investigate the molecular mechanisms of herb Rhodiola rosea L. radix	2018	Wenjuan Zhang, Ying Huai, Zhiping Miao, Chu Chen, Mohamed Shahen, Siddiq Ur Rahman, Mahmoud Alagawany, Mahmoud E. Abd El-Hack, Heping Zhao, Airong Qian	Xi’an JiaoTong University College of Medicine, Tanta University, Northwest A&F University, Zagazig University	Chinese Academy of Sciences Chemistry database, Chinese Herbal Drug Database, TCMSP	TCMSP	Gene ontology, KEGG	[113]
Systems pharmacology based strategy for Q-markers discovery of HuangQin decoction to attenuate intestinal damage	2018	Xiaomin Dai, Dongni Cui, Jing Wang, Wei Zhang, Zunjian Zhang, Fengguo Xu	China Pharmaceutical University, Shaanxi University of Chinese Medicine, Macau University of Science and Technology	TCMSP, TCMID	TCMSP, STITCH, Drugbank	.	[114]
Systems Pharmacology Dissection of Traditional Chinese Medicine Wen-Dan Decoction for Treatment of Cardiovascular Diseases	2018	Taohua Lan, Lulu Zhang, Yonghua Wang, Huanlin Wu, Danping Xu	Guangzhou University of Chinese Medicine, Northwest A&F University, Beijing University of Chinese Medicine	TCMSP	TCMSP	Gene ontology	[115]

Systems pharmacology uncover the mechanism of anti-non-small cell lung cancer for Hedyotis diffusa Willd	2018	Xing Su, Yueping Li, Meng Jiang, Jinglin Zhu, Chunli Zheng, Xuetong Chen, Jun Zhou, Yan Li, Wei Xiao, Yonghua Wang	Shihezi University, Northwest University, Northwest A&F University, Jiangsu Kanion Parmaceutical Corporation, Dalian University of Technology	TCMSP	TCMSP, WES	Gene ontology, KEGG	[116]
Systems pharmacology-based exploration reveals mechanisms of anti-steatotic effects of Jiang Zhi Granule on non-alcoholic fatty liver disease	2018	Yiyuan Zheng, Miao Wang, Peiyong Zheng, Xudong Tang, Guang Ji	Shanghai University of Traditional Chinese Medicine, Xiyuan Hospital of China Academy of Chinese Medical Sciences	TCMSP	TCMSP	OMIM, TTD, GeneCards	[117]
Molecular targets of Chinese herbs: a clinical study of metastatic colorectal cancer based on network pharmacology.	2018	Hongxu Zhu, Jian Hao, Yangyang Niu, Dan Liu, Dan Chen, Xiongzhi Wu	Tianjin Medical University	TCMSP	TCMSP	.	[118]
Screening and analysis of key active constituents in Guanxinshutong capsule using mass spectrum and integrative network pharmacology.	2018	Feng Liu, Xia Du, PeiRong Liu, YuHong Sun, YanMin Zhang	Xian Jiaotong University, Shannxi Academy of Traditionnal Chinese Medicine, Northwest University	LC-MS	CSDT	KEGG	[119]
Systems pharmacology dissection of the anti-stroke mechanism for the Chinese traditional medicine Xing-Nao-Jing.	2018	Yuhua Chen, Yue Sun, Wende Li, Hong Wei, Tianlin Long, Hua Li, Quanhua Xu, Wei Liu	Peihua University, PLA Army General Hospital, Shaanxi Fourth People's Hospital, Bijie First People's Hospital	TCMSP	TCMSP, SEA	TTD, PharmGkb, DisGeNET, Gene ontology	[120]

A Computational Systems Pharmacology Approach to Investigate Molecular Mechanisms of Herbal Formula Tian-Ma-Gou-Teng-Yin for Treatment of Alzheimer's Disease	2018	Tianduanyi Wang, Zengrui Wu, Lixia Sun, Weihua Li, Guixia Liu, Yun Tang	East China University of Science and Technology	TCMSP, TCMID, TCM Database@taiwan	bSDTNBI	HuGE, KEGG disease, OMIM, PharmGkb, KEGG, Gene ontology	[121]
A Network Pharmacology Analysis to Explore the Effect of Astragali Radix-Radix Angelica Sinensis on Traumatic Brain Injury	2018	Genggeng Xie, Weijun Peng, Pengfei Li, Zian Xia, Yuanyuan Zhong, Feng He, Yimingaji Tulake, Dandan Feng, Yang Wang, Zhihua Xing	Central South University, Xinjiang Production and Construction Corps Third Division Hospital	TCMSP	TCMSP, Literature mining	KEGG, Gene ontology, TTD, OMIM	[122]
A network pharmacology approach to explore the mechanisms of Erxian decoction in polycystic ovary syndrome	2018	Lihong Liu, Bo Du, Haiying Zhang, Xiaofei Guo, Zheng Zhou, Aihui Xiu, Chang Liu, Shiyu Su, Hao Ai	Jinzhou Medical University	TCMSP	STITCH	NCBI Gene database, Gene ontology, KEGG	[123]
A New Strategy to Uncover the Anticancer Mechanism of Chinese Compound Formula by Integrating Systems Pharmacology and Bioinformatics	2018	Yifei Dai, Liang Sun, Weijie Qiang	China Academy of Chinese Medical Sciences, The China Institute for History of Medicine and Medical Literature	TCMSP, TCMID, TCM Database@Taiwan	TCMSP	KEGG, Drugbank	[124]
Integrating Network Pharmacology and Metabolomics Study on Anti-rheumatic Mechanisms and Antagonistic Effects	2018	Jian Zuo, Xin Wang, Yang Liu, Jing Ye, Qingfei Liu, Yan Li, Shao Li	Wannan Medical College, Tsinghua University	Literature mining	drugCIPHER	Gene ontology, KEGG	[125]

Against Methotrexate-Induced Toxicity of Qing-Luo-Yin							
Identification of Pharmacokinetic Markers for Guanxin Danshen Drop Pills in Rats by Combination of Pharmacokinetics, Systems Pharmacology, and Pharmacodynamic Assays	2018	Hong Yao, Xiaomei Huang, Yunjiao Xie, Xuliang Huang, Yijun Ruan, Xinhua Lin, Liying Huang, Peiying Shi	Fujian Medical University, Fujian Agriculture and Forestry University	HPLC	PharmMapper	.	[126]
Anti-inflammatory effects of Zhishi and Zhiqiao revealed by network pharmacology integrated with molecular mechanism and metabolomics studies	2018	Siyu Zhao, Zhenli Liu, Menglei Wang, Dan He, Linlin Liu, Yisong Shu, Zhiqian Song, Hui Li, Yuanyan Liu, Aiping Lu	Beijing University of Chinese Medicine, China Academy of Chinese Medical Sciences, Hong Kong Baptist University	RRLC-QqQ-MS	PubChem, Drugbank, String, PharmMapper	KEGG, HMDB	[127]
Bioinformatics Based Therapeutic Effects of Sinomenium Acutum	2018	Yuyan Li, Guang Zheng, Liang Liu	Macau University of Science and Technology, Shenzhen Traditional Chinese Medicine Hospital, Lanzhou University	Chinese Pharmacopoeia, Chinese Medicine Dictionaries, Traditional Chinese Medicine Database	PubChem	IPA, IPKB	[128]
Effects and mechanisms of Danshen-Shanzha herb-pair for atherosclerosis treatment using network pharmacology and	2018	Jianyong Zhang, Rixin Liang, Lan Wang, Bin Yang	China Academy of Chinese Medical Sciences	HPLC	PharmMapper	TTD, PharmGkb, Drugbank, KEGG	[129]

experimental pharmacology							
Effects of Qijian mixture on type 2 diabetes assessed by metabonomics, gut microbiota and network pharmacology	2018	Kuo Gao, Ran Yang, Jian Zhang, Zhiyong Wang, Caixia Jia, Feilong Zhang, Shaojing Li, Jinping Wang, Ghulam Murtaza, Hua Xie, Huihui Zhao, Wei Wang, Jianxin Chen	Beijing University of Chinese Medicine, China Academy of Chinese Medical Sciences, FengNing Chinese Medicine Hospital	UPLC	STITCH	KEGG	[130]
Integrated Network Pharmacology and Metabolomics Analysis of the Therapeutic Effects of Zi Dian Fang on Immune Thrombocytopenic Purpura	2018	Yubo Li, Yamei Li, Wenliang Lu, Hongbin Li, Yuming Wang, Houmin Luo, Yuanyuan Wu, Wenying Dong, Gang Bai, Yanjun Zhang	Tianjin University of Traditional Chinese Medicine, Tasly Pharmaceutical Group, Nankai University	UPLC	PharmMapper	KEGG	[131]
Integration of transcriptomics, proteomics, metabolomics and systems pharmacology data to reveal the therapeutic mechanism underlying Chinese herbal Bufei Yishen formula for the treatment of chronic obstructive pulmon	2018	Peng Zhao, Jiansheng Li, Liping Yang, Ya Li, Yange Tian, Suyun Li	Henan University of Chinese Medicine	HPLC	Microarray	KEGG, Gene ontology	[132]
Multifactorial Modes of Action of Arsenic Trioxide in Cancer Cells as Analyzed by Classical and Network	2018	Mona Dawood, Sami Hamdoun, Thomas Efferth	Johannes Gutenberg University	.	Microarray	IPA	[133]

Pharmacology							
Network pharmacology study reveals energy metabolism and apoptosis pathways-mediated cardioprotective effects of Shenqi Fuzheng	2018	Jie Liao, Cui Hao, Wenhua Huang, Xin Shao, Yangang Song, Liangfeng Liu, Ni Ai, Xiaohui Fan	Zhejiang University, Livzon Pharmaceutical Group Inc.	.	Microarray	KEGG	[134]
Network Pharmacology to Unveil the Biological Basis of Health-Strengthening Herbal Medicine in Cancer Treatment	2018	Jiahui Zheng, Min Wu, Haiyan Wang, Shasha Li, Xin Wang, Yan Li, Dong Wang, Shao Li	Tsinghua University, Chinese Academy of Medical Sciences, Peking Union Medical College	.	drugCIPHER	KEGG, OMIM	[135]
Network pharmacology-based prediction of the active ingredients, potential targets, and signaling pathways in compound Lian-Ge granules for treatment of diabetes	2018	Jintao Xue, Yongli Shi, Chunyan Li, Huijie Song	Xinxiang Medical University	UPLC	PharmMapper, Drugbank	KEGG, Gene ontology	[136]
Network pharmacology-based strategy to investigate pharmacological mechanisms of Zuojinwan for treatment of gastritis	2018	Guohua Yu, Wubin Wang, Xu Wang, Meng Xu, Lili Zhang, Lei Ding, Rui Guo, Yuanyuan Shi	Beijing University of Chinese Medicine	TCMSP	TCMSP, STITCH	Drugbank, OMIM, KEGG	[137]
Network Pharmacology-Based Validation of Caveolin-1 as a Key Mediator of Ai Du Qing Inhibition of Drug	2018	Neng Wang, Bowen Yang, Xiaotong Zhang, Shengqi Wang, Yifeng Zheng, Xiong Li, Shan Liu, Hao Pan, Yingwei	Guangzhou University of Chinese Medicine, The Second Affiliated Hospital of Guangzhou University	TCMSP, TCMID	Microarray	Gene ontology, KEGG	[138]

Resistance in Breast Cancer		Li, Zhujuan Huang, Fengxue Zhang, Zhiyu Wang	of Chinese Medicine, Guangzhou University of Chinese Medicine				
Systems pharmacology approach reveals the antiinflammatory effects of Ampelopsis grossedentata on dextran sodium sulfate-induced colitis	2018	Youlan Chen, Yali Zhang, Yancheng Dai, Zhipeng Tang	Shanghai University of Traditional Chinese Medicine	TCMSP	TCMSP	Genetic Association database, TTD	[139]
Systems Pharmacology-Based Approach of Connecting Disease Genes in Genome-Wide Association Studies with Traditional Chinese Medicine	2018	Jihye Kim, Minjae Yoo, Jimin Shin, Hyunmin Kim, Jaewoo Kang, Aik Choon Tan	University of Colorado Anschutz Medical Campus, Korea University	.	Microarray	GWAS	[140]
Systems pharmacology-based investigation of Sanwei Ganjiang Prescription; related mechanisms in liver injury	2018	Yunxia Luo, Xinyue Wang, Yujie Huang, Shuhuan Fang, Jun Wu, Yongbin Zhang, Tianqin Xiong, Cong Yang, Jiangang Shen, Chuanlan Sang, Qi Wang, Jiansong Fang	Guangzhou University of Chinese Medicine, Sun Yat-sen University, The University of Hong Kong	TCMSP	bSDTNBI	Gene ontology, KEGG	[141]
Uncovering the anticancer mechanism of Compound Kushen Injection against HCC by integrating quantitative analysis, network analysis and experimental validation	2018	Li Gao, Kexin Wang, Yuzhi Zhou, Jiansong Fang, Xuemei Qin, Guanhua Du	Shanxi University, Guangzhou University of Chinese Medicine, Chinese Academy of Medical Sciences, Peking Union Medical College	UPLC	ChemMapper, HIT	OncoDB.HCC, Liverome, KEGG,	[142]

Unveiling Active Constituents and Potential Targets Related to the Hematinic Effect of Steamed Panax notoginseng Using Network Pharmacology Coupled With Multivariate Data Analyses	2018	Yin Xiong, Yupiao Hu, Lijuan Chen, Zejun Zhang, Yiming Zhang, Ming Niu, Xiuming Cui	Kunming University of Science and Technology, 302 Military Hospital of China	HPLC	PharmMapper	OMIM, KEGG	[143]
Discovery of the mechanisms and major bioactive compounds responsible for the protective effects of Gualou Xiebai Decoction on coronary heart disease by network pharmacology analysis.	2018	Chong Li, WeiYang Zhang, Yang Yu, ChunSong Cheng, JingYan Han, XinSheng Yao, Hua Zhou	Macau University of Science and Technology, Guangzhou University of Chinese Medicine	UPLC	PharmMapper	Drugbank, TTD	[144]
TCMAnalyzer: A Chemo- and Bioinformatics Web Service for Analyzing Traditional Chinese Medicine.	2018	Zhihong Liu, Jiewen Du, Xin Yan, Jiali Zhong, Lu Cui, Jinyuan Lin, Lizhu Zeng, Peng Ding, Chen Pin, Xinxin Zhou, Huihao Zhou, Qiong Gu, Jun Xu	Sun Yat-sen University, Guangzhou University of Chinese Medicine	Chinese Pharmacopoeia	ChEMBL	TTD	[145]
Uncovering the Mechanisms of Chinese Herbal Medicine (MaZiRenWan) for Functional Constipation by Focused Network	2018	Tao Huang, Ziwan Ning, Dongdong Hu, Man Zhang, Ling Zhao, Chengyuan Lin, Linda-L.D. Zhong, Zhijun Yang, Hongxi Xu, Zhaoxiang Bian	Hong Kong Baptist University, Shanghai University of Traditional Chinese Medicine	BindingDB	MOST method	Pubmed	[146]

Pharmacology Approach.							
Network pharmacology-based identification of major component of Angelica sinensis and its action mechanism for the treatment of acute myocardial infarction.	2018	Xiaowei Niu, Jingjing Zhang, Jinrong Ni, Runqing Wang, Weiqiang Zhang, Shaobo Sun, Yu Peng, Ming Bai, Zheng Zhang	Lanzhou University, Gansu University of Chinese Medicine	TCMSP, TCM-mesh, BATMAN-TCM	TCMSP, TCM-mesh, BATMAN-TCM	TTD, GAD, PharmGkb, OMIM	[147]

Supplementary Table 2. Providing information and their types of methods employed in THM-NP studies

Name	Herb-Compound Network Construction	Compound-Target Network Construction				Target Interpretation		
		Chemogenomic Approach	Docking Simulation Approach	Ligand-Based Approach	Others	Biological Process	Pathway	Disease
AlzhCPI				○				
BATMAN-TCM	○	○				○	○	○
BindingDB	○							
Bio database								○
bSDTNBI		○						
CADGene								○
CHDD	○							
ChEMBL					○			
Chemistry database of Shanghai Institute of Organic Chemistry	○							
ChemMapper				○				
Chinese Academy of Science	○							
Chinese Academy of Sciences Chemistry	○							
Chinese Herbal Drug Database	○							
Chinese Medicine Dictionaries	○							
Chinese Natural Product Database	○							
Chinese Pharmacopoeia	○							

CNKI		○	○
Comparative Toxicogenomics Database		○	
Comprehensive natural products in TCM	○		
Cross-species drug-target model (CSDT)		○	
DisGeNET			○
Dr. Duke's phytochemical and ethnobotanical database	○		
Drugbank		○	○
drugCIPHER		○	
FDA orange book			○
GAD			○
Gene ontology			○
GeneCards			○
Genetic Association database			○
GWAS			○
Handbook of the Chemical Constutuents in Chinese Cerb Original Plants	○		
HerBiomap	○		
HIT		○	○
HMDB			○
HPLC	○		
HuGE			○
IPA			○
IPKB			○

KEGG						○	
KEGG disease							○
Korean traditional knowledge portal database	○						
LC-MS	○						
Literature mining	○			○			○
Liverome							○
MalaCards							○
Medchem				○			
Medicinal Plants Database	○						
Metadrug				○			
Microarray				○			
Molecular docking		○					
Molecule Annotation System					○		
MOST method			○				
NCBI Gene database							○
NMR	○						
OMIM							○
OncoDB.HCC							○
Organchem	○						
PANTHER					○		
Pharmacopoeia of the People's Republic of China	○						
PharmGkb							○
PharmMapper			○				
Potential drug target database							○
Potential Drug Target Database							○

PubChem		○	○
PubMed		○	○
QSAR		○	
RCSB PDB			○
Reaxys	○		
RNA-seq		○	
RRLC-QqQ-MS	○		
SciFinder	○	○	
SEA		○	
STITCH		○	
String		○	
SwissTargetPrediction		○	
T2D@ZJU			○
T2D-Db			○
Tanimoto coefficient		○	
TarNet		○	
TCM Database@taiwan	○		
TCMGeneDIT	○	○	
TCMID	○	○	
TCM-ID	○		
TCM-mesh		○	○
TCMN	○		
TCM-PTD	○	○	
TCMSP	○	○	○
T-HOD			○
TimTec plant extracts database	○		
Traditional Chinese Medicine Database	○		

TTD		○	○
UPLC	○		
UTRI			○
VIP database			○
Wangfang Database			○
WES		○	

References

1. Gu, J.; Zhang, H.; Chen, L.; Xu, S.; Yuan, G.; Xu, X. Drug-target network and polypharmacology studies of a Traditional Chinese Medicine for type II diabetes mellitus. *Comput. Biol. Chem.* **2011**.
2. Ye, H.Z.; Zheng, C.S.; Xu, X.J.; Wu, M.X.; Liu, X.X. Potential synergistic and multitarget effect of herbal pair Chuanxiong Rhizome-Paeonia Albifora Pall on osteoarthritis disease: A computational pharmacology approach. *Chin. J. Integr. Med.* **2011**.
3. Li, X.; Xu, X.; Wang, J.; Yu, H.; Wang, X.; Yang, H.; Xu, H.; Tang, S.; Li, Y.; Yang, L.; et al. A System-Level Investigation into the Mechanisms of Chinese Traditional Medicine: Compound Danshen Formula for Cardiovascular Disease Treatment. *PLoS One* **2012**.
4. Li, B.; Xu, X.; Wang, X.; Yu, H.; Li, X.; Tao, W.; Wang, Y.; Yang, L. A systems biology approach to understanding the mechanisms of action of chinese herbs for treatment of cardiovascular disease. *Int. J. Mol. Sci.* **2012**.
5. Wang, X.; Xu, X.; Tao, W.; Li, Y.; Wang, Y.; Yang, L. A Systems Biology Approach to Uncovering Pharmacological Synergy in Herbal Medicines with Applications to Cardiovascular Disease. *Evidence-Based Complement. Altern. Med.* **2012**.
6. Ye, L.; He, Y.; Ye, H.; Liu, X.P.; Yang, L.L.; Cao, Z.W.; Tang, K.L. Pathway-pathway network-based study of the therapeutic mechanisms by which salvianolic acid B regulates cardiovascular diseases. *Chinese Sci. Bull.* **2012**.
7. Zhao, J.; Yang, P.; Li, F.; Tao, L.; Ding, H.; Rui, Y.; Cao, Z.; Zhang, W. Therapeutic Effects of Astragaloside IV on Myocardial Injuries: Multi-Target Identification and Network Analysis. *PLoS One* **2012**.
8. Jiang, M.; Lu, C.; Chen, G.; Xiao, C.; Zha, Q.; Niu, X.; Chen, S.; Lu, A. Understanding the Molecular Mechanism of Interventions in Treating Rheumatoid Arthritis Patients with Corresponding Traditional Chinese Medicine Patterns Based on Bioinformatics Approach. *Evidence-Based Complement. Altern. Med.* **2012**.
9. Yao, Y.; Zhang, X.; Wang, Z.; Zheng, C.; Li, P.; Huang, C.; Tao, W.; Xiao, W.; Wang, Y.; Huang, L.; et al. Deciphering the combination principles of Traditional Chinese Medicine from a systems pharmacology perspective based on Ma-huang Decoction. *J. Ethnopharmacol.* **2013**.
10. Tao, W.; Xu, X.; Wang, X.; Li, B.; Wang, Y.; Li, Y.; Yang, L. Network pharmacology-based prediction of the active ingredients and potential targets of Chinese herbal Radix Curcumae formula for application to cardiovascular disease. *J. Ethnopharmacol.* 2013.
11. Liu, H.; Wang, J.; Zhou, W.; Wang, Y.; Yang, L. Systems approaches and polypharmacology for drug discovery from herbal medicines: An example using licorice. *J. Ethnopharmacol.* **2013**.
12. Wang, X.; Xu, X.; Li, Y.; Li, X.; Tao, W.; Li, B.; Wang, Y.; Yang, L. Systems pharmacology uncovers Janus functions of botanical drugs: Activation of host defense system and inhibition of influenza virus replication. *Integr. Biol. (United Kingdom)* **2013**.

13. Pei, L.; Bao, Y.; Liu, S.; Zheng, J.; Chen, X. Material Basis of Chinese Herbal Formulas Explored by Combining Pharmacokinetics with Network Pharmacology. *PLoS One* **2013**.

14. Wu, L.; Wang, Y.; Nie, J.; Fan, X.; Cheng, Y. A Network Pharmacology Approach to Evaluating the Efficacy of Chinese Medicine Using Genome-Wide Transcriptional Expression Data. *Evidence-Based Complement. Altern. Med.* **2013**.

15. Zhang, Y.; Wang, D.; Tan, S.; Xu, H.; Liu, C.; Lin, N. A Systems Biology-Based Investigation into the Pharmacological Mechanisms of Wu Tou Tang Acting on Rheumatoid Arthritis by Integrating Network Analysis. *Evidence-Based Complement. Altern. Med.* **2013**.

16. Zhang, B.; Wang, X.; Li, S. An Integrative Platform of TCM Network Pharmacology and Its Application on a Herbal Formula, Qing-Luo-Yin . *Evidence-Based Complement. Altern. Med.* **2013**.

17. Zheng, C.S.; Xu, X.J.; Ye, H.Z.; Wu, G.W.; Li, X.H.; Xu, H.F.; Liu, X.X. Network pharmacology-based prediction of the multi-target capabilities of the compounds in Taohong Siwu decoction, and their application in osteoarthritis. *Exp. Ther. Med.* **2013**.

18. Dai, W.; Chen, J.; Lu, P.; Gao, Y.; Chen, L.; Liu, X.; Song, J.; Xu, H.; Chen, D.; Yang, Y.; et al. Pathway Pattern-based prediction of active drug components and gene targets from H1N1 influenza's treatment with maxingshigan-yinqiaosan formula. *Mol. Biosyst.* **2013**.

19. Ma, S.; Feng, C.; Zhang, X.; Dai, G.; Li, C.; Cheng, X.; Liu, P.; Ju, W.; Yu, H. The multi-target capabilities of the compounds in a TCM used to treat sepsis and their in silico pharmacology. *Complement. Ther. Med.* **2013**.

20. Sun, Y.; Zhu, R.; Ye, H.; Tang, K.; Zhao, J.; Chen, Y.; Liu, Q.; Cao, Z. Towards a bioinformatics analysis of anti-alzheimer's herbalmedicines from a target network perspective. *Brief. Bioinform.* **2013**.

21. Li, Y.; Han, C.; Wang, J.; Xiao, W.; Wang, Z.; Zhang, J.; Yang, Y.; Zhang, S.; Ai, C. Investigation into the mechanism of Eucommia ulmoides Oliv. based on a systems pharmacology approach. *J. Ethnopharmacol.* **2014**.

22. Li, B.; Tao, W.; Zheng, C.; Shar, P.A.; Huang, C.; Fu, Y.; Wang, Y. Systems pharmacology-based approach for dissecting the addition and subtraction theory of traditional Chinese medicine: An example using Xiao-Chaihu-Decoction and Da-Chaihu-Decoction. *Comput. Biol. Med.* **2014**.

23. Shi, S.H.; Cai, Y.P.; Cai, X.J.; Zheng, X.Y.; Cao, D.S.; Ye, F.Q.; Xiang, Z. A network pharmacology approach to understanding the mechanisms of action of traditional medicine: Bushenhuoxue formula for treatment of chronic kidney disease. *PLoS One* **2014**.

24. Liang, X.; Li, H.; Li, S. A novel network pharmacology approach to analyse traditional herbal formulae: The Liu-Wei-Di-Huang pill as a case study. *Mol. Biosyst.* **2014**.

25. Xu, H.; Zhang, Y.; Lei, Y.; Gao, X.; Zhai, H.; Lin, N.; Tang, S.; Liang, R.; Ma, Y.; Li, D.; et al. A systems biology-based approach to uncovering the molecular mechanisms underlying the effects of dragon's blood tablet in colitis, involving the integration of chemical analysis, ADME prediction,

and network pharmacology. *PLoS One* **2014**.

26. Yang, H.; Zhang, W.; Huang, C.; Zhou, W.; Yao, Y.; Wang, Z.; Li, Y.; Xiao, W.; Wang, Y. A novel systems pharmacology model for herbal medicine injection: A case using reduning injection. *BMC Complement. Altern. Med.* **2014**.

27. Li, H.; Zhao, L.; Zhang, B.; Jiang, Y.; Wang, X.; Guo, Y.; Liu, H.; Li, S.; Tong, X. A Network Pharmacology Approach to Determine Active Compounds and Action Mechanisms of Ge-Gen-Qin-Lian Decoction for Treatment of Type 2 Diabetes. *Evidence-Based Complement. Altern. Med.* **2014**.

28. Li, X.; Wu, L.; Liu, W.; Jin, Y.; Chen, Q.; Wang, L.; Fan, X.; Li, Z.; Cheng, Y. A network pharmacology study of Chinese medicine QiShenYiQi to reveal its underlying multi-compound, multi-target, multi-pathway mode of action. *PLoS One* **2014**.

29. Gu, H.; Ma, L.; Ren, Y.; He, W.; Wang, Y.; Qiao, Y. Exploration of the mechanism of pattern-specific treatments in coronary heart disease with network pharmacology approach. *Comput. Biol. Med.* **2014**.

30. Wu, L.; Wang, Y.; Li, Z.; Zhang, B.; Cheng, Y.; Fan, X. Identifying roles of " Jun-Chen-Zuo-Shi" component herbs of QiShenYiQi formula in treating acute myocardial ischemia by network pharmacology. *Chinese Med. (United Kingdom)* **2014**.

31. Sheng, S.; Wang, J.; Wang, L.; Liu, H.; Li, P.; Liu, M.; Long, C.; Xie, C.; Xie, X.; Su, W. Network pharmacology analyses of the antithrombotic pharmacological mechanism of Fufang Xueshuantong Capsule with experimental support using disseminated intravascular coagulation rats. *J. Ethnopharmacol.* **2014**.

32. Zhang, X.; Gu, J.; Cao, L.; Li, N.; Ma, Y.; Su, Z.; Ding, G.; Chen, L.; Xu, X.; Xiao, W. Network pharmacology study on the mechanism of traditional Chinese medicine for upper respiratory tract infection. *Mol. Biosyst.* **2014**.

33. Ru, J.; Li, P.; Wang, J.; Zhou, W.; Li, B.; Huang, C.; Li, P.; Guo, Z.; Tao, W.; Yang, Y.; et al. TCMSP: A database of systems pharmacology for drug discovery from herbal medicines. *J. Cheminform.* **2014**, *6*, 1–6.

34. Zhao, F.; Guochun, L.; Yang, Y.; Shi, L.; Xu, L.; Yin, L. A network pharmacology approach to determine active ingredients and rationality of herb combinations of Modified-Simiaowan for treatment of gout. *J. Ethnopharmacol.* **2015**.

35. Luo, Y.; Wang, Q.; Zhang, Y. A systems pharmacology approach to decipher the mechanism of danggui-shaoyao-san decoction for the treatment of neurodegenerative diseases. *J. Ethnopharmacol.* **2016**.

36. Tang, F.; Tang, Q.; Tian, Y.; Fan, Q.; Huang, Y.; Tan, X. Network pharmacology-based prediction of the active ingredients and potential targets of Mahuang Fuzi Xixin decoction for application to allergic rhinitis. *J. Ethnopharmacol.* **2015**.

37. Wang, Y.; Zheng, C.; Huang, C.; Li, Y.; Chen, X.; Wu, Z.; Wang, Z.; Xiao, W.; Zhang, B. Systems Pharmacology Dissecting Holistic Medicine for

Treatment of Complex Diseases: An Example Using Cardiocerebrovascular Diseases Treated by TCM. *Evidence-Based Complement. Altern. Med.* **2015**.

38. Li, J.; Zhao, P.; Li, Y.; Tian, Y.; Wang, Y. Systems pharmacology-based dissection of mechanisms of Chinese medicinal formula Bufei Yishen as an effective treatment for chronic obstructive pulmonary disease. *Sci. Rep.* **2015**.

39. Xu, F.; Ding, Y.; Guo, Y.; Liu, B.; Kou, Z.; Xiao, W.; Zhu, J. Anti-osteoporosis effect of Epimedium via an estrogen-like mechanism based on a system-level approach. *J. Ethnopharmacol.* **2016**.

40. Jiang, X.; Lv, B.; Li, P.; Ma, X.; Wang, T.; Zhou, Q.; Wang, X.; Gao, X. Bioactivity-integrated UPLC/Q-TOF-MS of Danhong injection to identify NF-κB inhibitors and anti-inflammatory targets based on endothelial cell culture and network pharmacology. *J. Ethnopharmacol.* **2015**.

41. Xiang, Z.; Sun, H.; Cai, X.; Chen, D.; Zheng, X. The study on the material basis and the mechanism for anti-renal interstitial fibrosis efficacy of rhubarb through integration of metabonomics and network pharmacology. *Mol. Biosyst.* **2015**.

42. Zheng, C.S.; Fu, C.L.; Pan, C. Bin; Bao, H.J.; Chen, X.Q.; Ye, H.Z.; Ye, J.X.; Wu, G.W.; Li, X.H.; Xu, H.F.; et al. Deciphering the underlying mechanisms of Diesun Miaofang in traumatic injury from a systems pharmacology perspective. *Mol. Med. Rep.* **2015**.

43. Guo, Q.; Zhong, M.; Xu, H.; Mao, X.; Zhang, Y.; Lin, N. A Systems Biology Perspective on the Molecular Mechanisms Underlying the Therapeutic Effects of Buyang Huanwu Decoction on Ischemic Stroke. *Rejuvenation Res.* **2015**.

44. Huang, L.; Lv, Q.; Liu, F.; Shi, T.; Wen, C. A Systems Biology-Based Investigation into the Pharmacological Mechanisms of Sheng-ma-bie-jia-tang Acting on Systemic Lupus Erythematosus by Multi-Level Data Integration. *Sci. Rep.* **2015**.

45. Li, Y.; Li, R.; Ouyang, Z.; Li, S. Herb Network Analysis for a Famous TCM Doctor's Prescriptions on Treatment of Rheumatoid Arthritis. *Evidence-Based Complement. Altern. Med.* **2015**.

46. An, L.; Feng, F. Network Pharmacology-Based Antioxidant Effect Study of Zhi-Zi-Da-Huang Decoction for Alcoholic Liver Disease. *Evidence-Based Complement. Altern. Med.* **2015**.

47. Zhang, B.; Lu, C.; Bai, M.; He, X.; Tan, Y.; Bian, Y.; Xiao, C.; Zhang, G.; Lu, A.; Li, S. Tetramethylpyrazine identified by a network pharmacology approach ameliorates methotrexate-induced oxidative organ injury. *J. Ethnopharmacol.* **2015**.

48. Gao, L.; Wang, X.D.; Niu, Y.Y.; Duan, D.D.; Yang, X.; Hao, J.; Zhu, C.H.; Chen, D.; Wang, K.X.; Qin, X.M.; et al. Molecular targets of Chinese herbs: a clinical study of hepatoma based on network pharmacology. *Sci. Rep.* **2016**.

49. Pei, T.; Zheng, C.; Huang, C.; Chen, X.; Guo, Z.; Fu, Y.; Liu, J.; Wang, Y. Systematic understanding the mechanisms of vitiligo pathogenesis and its treatment by Qubaibabuqi formula. *J. Ethnopharmacol.* **2016**.

50. Zhang, W.; Tao, Q.; Guo, Z.; Fu, Y.; Chen, X.; Shar, P.A.; Shahen, M.; Zhu, J.; Xue, J.; Bai, Y.; et al. Systems Pharmacology Dissection of the Integrated Treatment for Cardiovascular and Gastrointestinal Disorders by Traditional Chinese Medicine. *Sci. Rep.* **2016**.

51. Zhou, W.; Wang, J.; Wu, Z.; Huang, C.; Lu, A.; Wang, Y. Systems pharmacology exploration of botanic drug pairs reveals the mechanism for treating different diseases. *Sci. Rep.* **2016**.

52. Liu, J.; Pei, T.; Mu, J.; Zheng, C.; Chen, X.; Huang, C.; Fu, Y.; Liang, Z.; Wang, Y. Systems Pharmacology Uncovers the Multiple Mechanisms of Xijiao Dihuang Decoction for the Treatment of Viral Hemorrhagic Fever. *Evidence-Based Complement. Altern. Med.* **2016**.

53. Liu, J.; Mu, J.; Zheng, C.; Chen, X.; Guo, Z.; Huang, C.; Fu, Y.; Tian, G.; Shang, H.; Wang, Y. Systems-Pharmacology Dissection of Traditional Chinese Medicine Compound Saffron Formula Reveals Multi-scale Treatment Strategy for Cardiovascular Diseases. *Sci. Rep.* **2016**.

54. Gao, L.; Hao, J.; Niu, Y.Y.; Tian, M.; Yang, X.; Zhu, C.H.; Ding, X.L.; Liu, X.H.; Zhang, H.R.; Liu, C.; et al. Network pharmacology dissection of multiscale mechanisms of herbal medicines in stage IV gastric adenocarcinoma treatment. *Med. (United States)* **2016**.

55. Shen, X.; Zhao, Z.; Luo, X.; Wang, H.; Hu, B.; Guo, Z. Systems Pharmacology Based Study of the Molecular Mechanism of SiNiSan Formula for Application in Nervous and Mental Diseases. *Evidence-Based Complement. Altern. Med.* **2016**.

56. Ke, Z.; Zhang, X.; Cao, Z.; Ding, Y.; Li, N.; Cao, L.; Wang, T.; Zhang, C.; Ding, G.; Wang, Z.; et al. Drug discovery of neurodegenerative disease through network pharmacology approach in herbs. *Biomed. Pharmacother.* **2016**.

57. Chen, L.; Lv, D.; Wang, D.; Chen, X.; Zhu, Z.; Cao, Y.; Chai, Y. A novel strategy of profiling the mechanism of herbal medicines by combining network pharmacology with plasma concentration determination and affinity constant measurement. *Mol. Biosyst.* **2016**.

58. Wang, C.H.; Zhong, Y.; Zhang, Y.; Liu, J.P.; Wang, Y.F.; Jia, W.N.; Wang, G.C.; Li, Z.; Zhu, Y.; Gao, X.M. A network analysis of the Chinese medicine Lianhua-Qingwen formula to identify its main effective components. *Mol. Biosyst.* **2016**.

59. Liu, H.; Zeng, L.; Yang, K.; Zhang, G. A network pharmacology approach to explore the pharmacological mechanism of xiaoyao powder on anovulatory infertility. *Evidence-based Complement. Altern. Med.* **2016**.

60. Liu, Z.; Guo, F.; Wang, Y.; Li, C.; Zhang, X.; Li, H.; Diao, L.; Gu, J.; Wang, W.; Li, D.; et al. BATMAN-TCM: A Bioinformatics Analysis Tool for Molecular mechANism of Traditional Chinese Medicine. *Sci. Rep.* **2016**, *6*, 1–11.

61. Liao, J.; Wei, B.; Chen, H.; Liu, Y.M.; Wang, J. Bioinformatics investigation of therapeutic mechanisms of xuesaitong capsule treating ischemic cerebrovascular rat model with comparative transcriptome analysis. *Am. J. Transl. Res.* **2016**.

62. Liang, H.; Ruan, H.; Ouyang, Q.; Lai, L. Herb-target interaction network analysis helps to disclose molecular mechanism of traditional Chinese medicine. *Sci. Rep.* **2016**.

63. Qi, Q.; Li, R.; Li, H.Y.; Cao, Y.B.; Bai, M.; Fan, X.J.; Wang, S.Y.; Zhang, B.; Li, S. Identification of the anti-tumor activity and mechanisms of nuciferine through a network pharmacology approach. *Acta Pharmacol. Sin.* **2016**.

64. Zhou, W.; Yuan, W.F.; Chen, C.; Wang, S.M.; Liang, S.W. Study on material base and action mechanism of compound Danshen dripping pills for treatment of atherosclerosis based on modularity analysis. *J. Ethnopharmacol.* **2016**.

65. Sun, J.; Zhang, L.; He, Y.; Zhang, K.; Wu, L.; Fan, Y.; Xie, Z. To unveil the molecular mechanisms of Qi and blood through systems biology-based investigation into Si-Jun-Zi-Tang and Si-Wu-Tang formulae. *Sci. Rep.* **2016**.

66. Ma, X.; Lv, B.; Li, P.; Jiang, X.; Zhou, Q.; Wang, X.; Gao, X. Identification of "Multiple Components-Multiple Targets-Multiple Pathways" Associated with Naoxintong Capsule in the Treatment of Heart Diseases Using UPLC/Q-TOF-MS and Network Pharmacology. *Evidence-Based Complement. Altern. Med.* **2016**.

67. Guo, S.; Li, P.; Fu, B.; Chuo, W.; Gao, K.; Zhang, W.; Wang, J.; Chen, J.; Wang, W. Systems-biology dissection of mechanisms and chemical basis of herbal formula in treating chronic myocardial ischemia. *Pharmacol. Res.* **2016**.

68. Wang, J.; Cui, C.; Fu, L.; Xiao, Z.; Xie, N.; Liu, Y.; Yu, L.; Wang, H.; Luo, B. Genomic expression profiling and bioinformatics analysis on diabetic nephrology with ginsenoside Rg3. *Mol. Med. Rep.* **2016**.

69. Wang, J.; Li, Y.; Yang, Y.; Du, J.; Zhao, M.; Lin, F.; Zhang, S.; Wang, B. Systems Pharmacology Dissection of Multiscale Mechanisms of Action for Herbal Medicines in Treating Rheumatoid Arthritis. *Mol. Pharm.* **2017**.

70. Zeng, L.; Yang, K.; Ge, J. Uncovering the Pharmacological Mechanism of Astragalus Salvia Compound on Pregnancy-Induced Hypertension Syndrome by a Network Pharmacology Approach. *Sci. Rep.* **2017**.

71. Tao, L.-J.; Chen, W.-T.; Jing, L.; Ji, Q.; Ren, J.-L. A network pharmacology approach to establish the pharmacological mechanism of JiaWeiXianJiTang on inflammatory bowel disease. *Biomed. Reports* **2017**.

72. Tang, H.C.; Huang, H.J.; Lee, C.C.; Chen, C.Y.C. Network pharmacology-based approach of novel traditional Chinese medicine formula for treatment of acute skin inflammation in silico. *Comput. Biol. Chem.* **2017**.

73. Yu, G.; Zhang, Y.; Ren, W.; Dong, L.; Li, J.; Geng, Y.; Zhang, Y.; Li, D.; Xu, H.; Yang, H. Network pharmacology-based identification of key pharmacological pathways of yin–huang–qing–fei capsule acting on chronic bronchitis. *Int. J. COPD* **2016**.

74. Wang, D.; Gu, J.; Zhu, W.; Luo, F.; Chen, L.; Xu, X.; Lu, C. PDTCM: a systems pharmacology platform of traditional Chinese medicine for psoriasis. *Ann. Med.* **2017**.

75. Kong, Q.; Ma, Y.; Yu, J.; Chen, X. Predicted molecular targets and pathways for germacrone, curdione, and furanodiene in the treatment of breast

cancer using a bioinformatics approach. *Sci. Rep.* **2017**.

76. Li, F.; Zhang, Y.; Zeng, D.; Xia, Y.; Fan, X.; Tan, Y.; Kou, J.; Yu, B. The combination of three components derived from sheng maisan protects myocardial ischemic diseases and inhibits oxidative stress via modulating MAPKs and JAK2-STAT3 signaling pathways based on bioinformatics approach. *Front. Pharmacol.* **2017**.

77. Hong, M.; Zhang, Y.; Li, S.; Tan, H.Y.; Wang, N.; Mu, S.; Hao, X.; Feng, Y. A network pharmacology-based study on the hepatoprotective effect of fructus schisandrae. *Molecules* **2017**.

78. Hong, M.; Li, S.; Wang, N.; Tan, H.Y.; Cheung, F.; Feng, Y. A biomedical investigation of the hepatoprotective effect of radix salviae miltiorrhizae and network pharmacology-based prediction of the active compounds and molecular targets. *Int. J. Mol. Sci.* **2017**.

79. Li, Y.; Wang, J.; Lin, F.; Yang, Y.; Chen, S.S. A methodology for cancer therapeutics by systems pharmacology-based analysis: A case study on breast cancer-related traditional Chinese medicines. *PLoS One* **2017**.

80. Zeng, L.; Yang, K. Exploring the pharmacological mechanism of Yanghe Decoction on HER2-positive breast cancer by a network pharmacology approach. *J. Ethnopharmacol.* **2017**.

81. Huang, J.; Cheung, F.; Tan, H.Y.; Hong, M.; Wang, N.; Yang, J.; Feng, Y.; Zheng, Q. Identification of the active compounds and significant pathways of yinchenhao decoction based on network pharmacology. *Mol. Med. Rep.* **2017**.

82. Huang, J.; Tang, H.; Cao, S.; He, Y.; Feng, Y.; Wang, K.; Zheng, Q. Molecular Targets and Associated Potential Pathways of Danlu Capsules in Hyperplasia of Mammary Glands Based on Systems Pharmacology. *Evidence-Based Complement. Altern. Med.* **2017**.

83. Park, S.Y.; Park, J.H.; Kim, H.S.; Lee, C.Y.; Lee, H.J.; Kang, K.S.; Kim, C.E. Systems-level mechanisms of action of Panax ginseng: a network pharmacological approach. *J. Ginseng Res.* 2018.

84. Mao, Y.; Hao, J.; Jin, Z.; Niu, Y.; Yang, X.; Liu, D.; Cao, R.; Wu, X. Network pharmacology-based and clinically relevant prediction of the active ingredients and potential targets of Chinese herbs in metastatic breast cancer patients. *Oncotarget* **2017**.

85. Wang, N.; Zheng, Y.; Gu, J.; Cai, Y.; Wang, S.; Zhang, F.; Chen, J.; Situ, H.; Lin, Y.; Wang, Z. Network-pharmacology-based validation of TAMS/CXCL-1 as key mediator of XIAOPI formula preventing breast cancer development and metastasis. *Sci. Rep.* **2017**.

86. Wang, N.; Zhao, G.; Zhang, Y.; Wang, X.; Zhao, L.; Xu, P.; Shou, D. A Network Pharmacology Approach to Determine the Active Components and Potential Targets of Curculigo Orchioides in the Treatment of Osteoporosis. *Med. Sci. Monit.* **2017**.

87. Tan, W.; Wu, T.; Yang, C.; Cai, H.; Fang, S.; Chen, Y.; Wang, Q. Network pharmacology-based study on the mechanism of action for herbal medicines in Alzheimer treatment. *J. Ethnopharmacol.* **2017**.

88. Zeng, L.; Yang, K.; Liu, H.; Zhang, G. A network pharmacology approach to investigate the pharmacological effects of Guizhi Fuling Wan on uterine fibroids. *Exp. Ther. Med.* **2017**.

89. Zhang, Y.; Mao, X.; Su, J.; Geng, Y.; Guo, R.; Tang, S.; Li, J.; Xiao, X.; Xu, H.; Yang, H. A network pharmacology-based strategy deciphers the underlying molecular mechanisms of Qixuehe Capsule in the treatment of menstrual disorders. *Chinese Med. (United Kingdom)* **2017**.

90. Suo, T.; Liu, J.; Chen, X.; Yu, H.; Wang, T.; Li, C.; Wang, Y.; Wang, C.; Li, Z. Combining Chemical Profiling and Network Analysis to Investigate the Pharmacology of Complex Prescriptions in Traditional Chinese Medicine. *Sci. Rep.* **2017**.

91. HU, R.F.; SUN, X.B. Design of new traditional Chinese medicine herbal formulae for treatment of type 2 diabetes mellitus based on network pharmacology. *Chin. J. Nat. Med.* **2017**.

92. Xie, D.; Huang, L.; Zhao, G.; Yu, Y.; Gao, J.; Li, H.; Wen, C. Dissecting the Underlying Pharmaceutical Mechanism of Chinese Traditional Medicine Yun-Pi-Yi-Shen-Tong-Du-Tang Acting on Ankylosing Spondylitis through Systems Biology Approaches. *Sci. Rep.* **2017**.

93. Zhang, Y.Q.; Wang, C.; Guo, Q.Y.; Zhu, C.Y.; Yan, C.; Sun, D.N.; Xu, Q.H.; Lin, N. Molecular mechanisms of the analgesic action of Wu-tou Decoction on neuropathic pain in mice revealed using microarray and network analysis. *Acta Pharmacol. Sin.* **2018**.

94. Lyu, M.; Yan, C.L.; Liu, H.X.; Wang, T.Y.; Shi, X.H.; Liu, J.P.; Orgah, J.; Fan, G.W.; Han, J.H.; Wang, X.Y.; et al. Network pharmacology exploration reveals endothelial inflammation as a common mechanism for stroke and coronary artery disease treatment of Danhong injection. *Sci. Rep.* **2017**.

95. Wang, J.; Liu, R.; Liu, B.; Yang, Y.; Xie, J.; Zhu, N. Systems Pharmacology-based strategy to screen new adjuvant for hepatitis B vaccine from Traditional Chinese Medicine Ophiocordyceps sinensis. *Sci. Rep.* **2017**.

96. Li, H.; Liu, L.; Liu, C.; Zhuang, J.; Zhou, C.; Yang, J.; Gao, C.; Liu, G.; Lv, Q.; Sun, C. Deciphering Key Pharmacological Pathways of Qingdai Acting on Chronic Myeloid Leukemia Using a Network Pharmacology-Based Strategy. *Med. Sci. Monit.* **2018**.

97. Zhong, Y.; Luo, J.; Tang, T.; Li, P.; Liu, T.; Cui, H.; Wang, Y.; Huang, Z. Exploring Pharmacological Mechanisms of Xuefu Zhuyu Decoction in the Treatment of Traumatic Brain Injury via a Network Pharmacology Approach. *Evidence-Based Complement. Altern. Med.* **2018**.

98. Li, J.; Ma, X.; Liu, C.; Li, H.; Zhuang, J.; Gao, C.; Zhou, C.; Liu, L.; Wang, K.; Sun, C. Exploring the Mechanism of Danshen against Myelofibrosis by Network Pharmacology and Molecular Docking. *Evidence-Based Complement. Altern. Med.* **2018**.

99. Pang, H.Q.; Yue, S.J.; Tang, Y.P.; Chen, Y.Y.; Tan, Y.J.; Cao, Y.J.; Shi, X.Q.; Zhou, G.S.; Kang, A.; Huang, S.L.; et al. Integrated metabolomics and network pharmacology approach to explain possible action mechanisms of Xin-Sheng-Hua granule for treating anemia. *Front. Pharmacol.* **2018**.

100. Bing, Z.; Cheng, Z.; Shi, D.; Liu, X.; Tian, J.; Yao, X.; Zhang, J.; Wang, Y.; Yang, K. Investigate the mechanisms of Chinese medicine Fuzhengkangai towards EGFR mutation-positive lung adenocarcinomas by network pharmacology. *BMC Complement. Altern. Med.* **2018**.

101. Zhao, M.; Chen, Y.; Wang, C.; Xiao, W.; Chen, S.; Zhang, S.; Yang, L.; Li, Y. Systems pharmacology dissection of multi-scale mechanisms of action of Huo-Xiang-Zheng-qi formula for the treatment of gastrointestinal diseases. *Front. Pharmacol.* **2019**.

102. Dai, W.; Chen, H.-Y.; Chen, C.Y.-C. A Network Pharmacology-Based Approach to Investigate the Novel TCM Formula against Huntington's Disease and Validated by Support Vector Machine Model. *Evidence-Based Complement. Altern. Med.* **2018**.

103. Zhou, C.; Liu, L.; Zhuang, J.; Wei, J.; Zhang, T.; Gao, C.; Liu, C.; Li, H.; Si, H.; Sun, C. A Systems Biology-Based Approach to Uncovering Molecular Mechanisms Underlying Effects of Traditional Chinese Medicine Qingdai in Chronic Myelogenous Leukemia, Involving Integration of Network Pharmacology and Molecular Docking Technology. *Med. Sci. Monit.* **2018**.

104. Wang, J.B.; Cui, H.R.; Wang, R.L.; Zhang, C.E.; Niu, M.; Bai, Z.F.; Xu, G.H.; Li, P.Y.; Jiang, W.Y.; Han, J.J.; et al. A systems pharmacology-oriented discovery of a new therapeutic use of the TCM formula Liuweiwuling for liver failure. *Sci. Rep.* **2018**.

105. Chen, L.; Cao, Y.; Zhang, H.; Lv, D.; Zhao, Y.; Liu, Y.; Ye, G.; Chai, Y. Network pharmacology-based strategy for predicting active ingredients and potential targets of Yangxinshi tablet for treating heart failure. *J. Ethnopharmacol.* **2018**.

106. Gómez-Verjan, J.C.; Rivero-Segura, N.A.; Estrella-Parra, E.; Rincón-Heredia, R.; Madariaga-Mazón, A.; Flores-Soto, E.; González-Meljem, M.; Cerbón, M.; Reyes-Chilpa, R. Network Pharmacology Uncovers Anticancer Activity of Mammea-Type Coumarins from Calophyllum brasiliense. *Planta Med.* **2019**.

107. Park, M.; Park, S.-Y.; Lee, H.-J.; Kim, C.-E. A Systems-Level Analysis of Mechanisms of Platycodon grandiflorum Based on A Network Pharmacological Approach. *Molecules* **2018**.

108. Lee, A.Y.; Park, W.; Kang, T.W.; Cha, M.H.; Chun, J.M. Network pharmacology-based prediction of active compounds and molecular targets in Yijin-Tang acting on hyperlipidaemia and atherosclerosis. *J. Ethnopharmacol.* **2018**.

109. Lee, D.; Kim, C.E.; Park, S.Y.; Kim, K.O.; Hiep, N.T.; Lee, D.; Jang, H.J.; Lee, J.W.; Kang, K.S. Protective effect of artemisia argyi and its flavonoid constituents against contrast-induced cytotoxicity by iodixanol in LLC-PK1 cells. *Int. J. Mol. Sci.* **2018**.

110. Gong, B.; Kao, Y.; Zhang, C.; Sun, F.; Zhao, H. Systematic Investigation of Scutellariae Barbatae Herba for Treating Hepatocellular Carcinoma Based on Network Pharmacology. *Evidence-Based Complement. Altern. Med.* **2018**.

111. Xu, T.; Ma, C.; Fan, S.; Deng, N.; Lian, Y.; Tan, L.; Du, W.; Zhang, S.; Liu, S.; Ren, B.; et al. Systematic Understanding of the Mechanism of Baicalin against Ischemic Stroke through a Network Pharmacology Approach. *Evidence-Based Complement. Altern. Med.* **2018**.

112. Liu, J.; Liu, J.; Shen, F.; Qin, Z.; Jiang, M.; Zhu, J.; Wang, Z.; Zhou, J.; Fu, Y.; Chen, X.; et al. Systems pharmacology analysis of synergy of TCM: An example using saffron formula. *Sci. Rep.* **2018**.

113. Zhang, W.; Huai, Y.; Miao, Z.; Chen, C.; Shahen, M.; Rahman, S.U.; Alagawany, M.; El-Hack, M.E.A.; Zhao, H.; Qian, A. Systems pharmacology approach to investigate the molecular mechanisms of herb Rhodiola rosea L. radix. *Drug Dev. Ind. Pharm.* **2019**.

114. Dai, X. min; Cui, D. ni; Wang, J.; Zhang, W.; Zhang, Z. jian; Xu, F. guo Systems pharmacology based strategy for Q-markers discovery of HuangQin decoction to attenuate intestinal damage. *Front. Pharmacol.* **2018**.

115. Lan, T.-H.; Zhang, L.-L.; Wang, Y.-H.; Wu, H.-L.; Xu, D.-P. Systems Pharmacology Dissection of Traditional Chinese Medicine Wen-Dan Decoction for Treatment of Cardiovascular Diseases. *Evidence-Based Complement. Altern. Med.* **2018**.

116. Su, X.; Li, Y.; Jiang, M.; Zhu, J.; Zheng, C.; Chen, X.; Zhou, J.; Li, Y.; Xiao, W.; Wang, Y. Systems pharmacology uncover the mechanism of anti-non-small cell lung cancer for Hedyotis diffusa Willd. *Biomed. Pharmacother.* **2019**.

117. Zheng, Y.; Wang, M.; Zheng, P.; Tang, X.; Ji, G. Systems pharmacology-based exploration reveals mechanisms of anti-steatotic effects of Jiang Zhi Granule on non-alcoholic fatty liver disease. *Sci. Rep.* **2018**.

118. Zhu, H.; Hao, J.; Niu, Y.; Liu, D.; Chen, D.; Wu, X. Molecular targets of Chinese herbs: A clinical study of metastatic colorectal cancer based on network pharmacology. *Sci. Rep.* **2018**.

119. LIU, F.; DU, X.; LIU, P.R.; SUN, Y.H.; ZHANG, Y.M. Screening and analysis of key active constituents in Guanxinshutong capsule using mass spectrum and integrative network pharmacology. *Chin. J. Nat. Med.* **2018**.

120. Chen, Y.; Sun, Y.; Li, W.; Wei, H.; Long, T.; Li, H.; Xu, Q.; Liu, W. Systems pharmacology dissection of the anti-stroke mechanism for the Chinese traditional medicine Xing-Nao-Jing. *J. Pharmacol. Sci.* **2018**.

121. Wang, T.; Wu, Z.; Sun, L.; Li, W.; Liu, G.; Tang, Y. A computational systems pharmacology approach to investigate molecular mechanisms of herbal formula Tian-Ma-Gou-Teng-Yin for treatment of Alzheimer's disease. *Front. Pharmacol.* **2018**.

122. Xie, G.; Peng, W.; Li, P.; Xia, Z.; Zhong, Y.; He, F.; Tulake, Y.; Feng, D.; Wang, Y.; Xing, Z. A Network Pharmacology Analysis to Explore the Effect of Astragali Radix-Radix Angelica Sinensis on Traumatic Brain Injury . *Biomed Res. Int.* **2018**.

123. Liu, L.; Du, B.; Zhang, H.; Guo, X.; Zhou, Z.; Xiu, A.; Liu, C.; Su, S.; Ai, H. A network pharmacology approach to explore the mechanisms of Erxian decoction in polycystic ovary syndrome. *Chinese Med. (United Kingdom)* **2018**.

124. Dai, Y.; Sun, L.; Qiang, W. A New Strategy to Uncover the Anticancer Mechanism of Chinese Compound Formula by Integrating Systems Pharmacology and Bioinformatics. *Evidence-Based Complement. Altern. Med.* **2018**.

125. Zuo, J.; Wang, X.; Liu, Y.; Ye, J.; Liu, Q.; Li, Y.; Li, S. Integrating Network Pharmacology and Metabolomics Study on Anti-rheumatic Mechanisms and Antagonistic Effects Against Methotrexate-Induced Toxicity of Qing-Luo-Yin. *Front. Pharmacol.* **2018**.

126. Yao, H.; Huang, X.; Xie, Y.; Huang, X.; Ruan, Y.; Lin, X.; Huang, L.; Shi, P. Identification of Pharmacokinetic Markers for Guanxin Danshen Drop Pills in Rats by Combination of Pharmacokinetics, Systems Pharmacology, and Pharmacodynamic Assays. *Front. Pharmacol.* **2018**.

127. Zhao, S.; Liu, Z.; Wang, M.; He, D.; Liu, L.; Shu, Y.; Song, Z.; Li, H.; Liu, Y.; Lu, A. Anti-inflammatory effects of Zhishi and Zhiqiao revealed by network pharmacology integrated with molecular mechanism and metabolomics studies. *Phytomedicine* **2018**.

128. Li, Y. yan; Zheng, G.; Liu, L. Bioinformatics Based Therapeutic Effects of Sinomenium Acutum. *Chin. J. Integr. Med.* **2019**.

129. Zhang, J.; Liang, R.; Wang, L.; Yang, B. Effects and mechanisms of Danshen-Shanzha herb-pair for atherosclerosis treatment using network pharmacology and experimental pharmacology. *J. Ethnopharmacol.* **2019**.

130. Gao, K.; Yang, R.; Zhang, J.; Wang, Z.; Jia, C.; Zhang, F.; Li, S.; Wang, J.; Murtaza, G.; Xie, H.; et al. Effects of Qijian mixture on type 2 diabetes assessed by metabonomics, gut microbiota and network pharmacology. *Pharmacol. Res.* **2018**.

131. Li, Y.; Li, Y.; Lu, W.; Li, H.; Wang, Y.; Luo, H.; Wu, Y.; Dong, W.; Bai, G.; Zhang, Y. Integrated network pharmacology and metabolomics analysis of the therapeutic effects of Zi Dian Fang on immune thrombocytopenic purpura. *Front. Pharmacol.* **2018**.

132. Zhao, P.; Li, J.; Yang, L.; Li, Y.; Tian, Y.; Li, S. Integration of transcriptomics, proteomics, metabolomics and systems pharmacology data to reveal the therapeutic mechanism underlying Chinese herbal Bufei Yishen formula for the treatment of chronic obstructive pulmonary disease. *Mol. Med. Rep.* **2018**.

133. Dawood, M.; Hamdoun, S.; Efferth, T. Multifactorial modes of action of arsenic trioxide in cancer cells as analyzed by classical and network pharmacology. *Front. Pharmacol.* **2018**.

134. Liao, J.; Hao, C.; Huang, W.; Shao, X.; Song, Y.; Liu, L.; Ai, N.; Fan, X. Network pharmacology study reveals energy metabolism and apoptosis pathways-mediated cardioprotective effects of Shenqi Fuzheng. *J. Ethnopharmacol.* **2018**.

135. Zheng, J.; Wu, M.; Wang, H.; Li, S.; Wang, X.; Li, Y.; Wang, D.; Li, S. Network Pharmacology to Unveil the Biological Basis of Health-Strengthening Herbal Medicine in Cancer Treatment. *Cancers (Basel).* **2018**.

136. Xue, J.; Shi, Y.; Li, C.; Song, H. Network pharmacology-based prediction of the active ingredients, potential targets, and signaling pathways in compound Lian-Ge granules for treatment of diabetes. *J. Cell. Biochem.* **2019**.

137. Yu, G.; Wang, W.; Wang, X.; Xu, M.; Zhang, L.; Ding, L.; Guo, R.; Shi, Y. Network pharmacology-based strategy to investigate pharmacological mechanisms of Zuojinwan for treatment of gastritis. *BMC Complement. Altern. Med.* **2018**.

138. Wang, N.; Yang, B.; Zhang, X.; Wang, S.; Zheng, Y.; Li, X.; Liu, S.; Pan, H.; Li, Y.; Huang, Z.; et al. Network pharmacology-based validation of caveolin-1 as a key mediator of Ai du Qing inhibition of drug resistance in breast cancer. *Front. Pharmacol.* **2018**.

139. Chen, Y.L.; Zhang, Y.L.; Dai, Y.C.; Tang, Z.P. Systems pharmacology approach reveals the antiinflammatory effects ofAmpelopsis grossedentata on dextran sodium sulfateinduced colitis. *World J. Gastroenterol.* **2018**.

140. Kim, J.; Yoo, M.; Shin, J.; Kim, H.; Kang, J.; Tan, A.C. Systems Pharmacology-Based Approach of Connecting Disease Genes in Genome-Wide Association Studies with Traditional Chinese Medicine. *Int. J. Genomics* **2018**, *2018*, 1–11.

141. LUO, Y.X.; WANG, X.Y.; HUANG, Y.J.; FANG, S.H.; WU, J.; ZHANG, Y. Bin; XIONG, T.Q.; YANG, C.; SHEN, J.G.; SANG, C.L.; et al. Systems pharmacology-based investigation of Sanwei Ganjiang Prescription: related mechanisms in liver injury. *Chin. J. Nat. Med.* **2018**.

142. Gao, L.; Wang, K.X.; Zhou, Y.Z.; Fang, J.S.; Qin, X.M.; Du, G.H. Uncovering the anticancer mechanism of Compound Kushen Injection against HCC by integrating quantitative analysis, network analysis and experimental validation. *Sci. Rep.* **2018**.

143. Xiong, Y.; Hu, Y.; Chen, L.; Zhang, Z.; Zhang, Y.; Niu, M.; Cui, X. Unveiling Active Constituents and Potential Targets Related to the Hematinic Effect of Steamed Panax notoginseng Using Network Pharmacology Coupled With Multivariate Data Analyses. *Front. Pharmacol.* **2019**.

144. Li, C.; Zhang, W.Y.; Yu, Y.; Cheng, C.S.; Han, J.Y.; Yao, X.S.; Zhou, H. Discovery of the mechanisms and major bioactive compounds responsible for the protective effects of Gualou Xiebai Decoction on coronary heart disease by network pharmacology analysis. *Phytomedicine* **2019**.

145. Liu, Z.; Du, J.; Yan, X.; Zhong, J.; Cui, L.; Lin, J.; Zeng, L.; Ding, P.; Chen, P.; Zhou, X.; et al. TCMAnalyzer: A Chemo- and Bioinformatics Web Service for Analyzing Traditional Chinese Medicine. *J. Chem. Inf. Model.* **2018**.

146. Huang, T.; Ning, Z.; Hu, D.; Zhang, M.; Zhao, L.; Lin, C.; Zhong, L.L.D.; Yang, Z.; Xu, H.; Bian, Z. Uncovering the mechanisms of Chinese herbal medicine (MaZiRenWan) for functional constipation by focused network pharmacology approach. *Front. Pharmacol.* **2018**.

147. Niu, X.; Zhang, J.; Ni, J.; Wang, R.; Zhang, W.; Sun, S.; Peng, Y.; Bai, M.; Zhang, Z. Network pharmacology-based identification of major component of Angelica sinensis and its action mechanism for the treatment of acute myocardial infarction . *Biosci. Rep.* **2018**.
